# The genetics of phenotypic plasticity. XI. Joint evolution of plasticity and dispersal rate

**DOI:** 10.1002/ece3.327

**Published:** 2012-07-20

**Authors:** Samuel M Scheiner, Michael Barfield, Robert D Holt

**Affiliations:** 1 Division of Environmental Biology, National Science FoundationArlington, VA, 22230; 2 Department of Biology, University of FloridaGainesville, FL, 32611

**Keywords:** Dispersal, model, phenotypic plasticity, theory

## Abstract

In a spatially heterogeneous environment, the rate at which individuals move among habitats affects whether selection favors phenotypic plasticity or genetic differentiation, with high dispersal rates favoring trait plasticity. Until now, in theoretical explorations of plasticity evolution, dispersal rate has been treated as a fixed, albeit probabilistic, characteristic of a population, raising the question of what happens when the propensity to disperse and trait plasticity are allowed to evolve jointly. We examined the effects of their joint evolution on selection for plasticity using an individual-based computer simulation model. In the model, the environment consisted of a linear gradient of 50 demes with dispersal occurring either before or after selection. Individuals consisted of loci whose phenotypic expression either are affected by the environment (plastic) or are not affected (nonplastic), plus a locus determining the propensity to disperse. When dispersal rate and trait plasticity evolve jointly, the system tends to dichotomous outcomes of either high trait plasticity and high dispersal, or low trait plasticity and low dispersal. The outcome strongly depended on starting conditions, with high trait plasticity and dispersal favored when the system started at high values for either trait plasticity or dispersal rate (or both). Adding a cost of plasticity tended to drive the system to genetic differentiation, although this effect also depended on initial conditions. Genetic linkage between trait plasticity loci and dispersal loci further enhanced this strong dichotomy in evolutionary outcomes. All of these effects depended on organismal life history pattern, and in particular whether selection occurred before or after dispersal. These results can explain why adaptive trait plasticity is less common than might be expected.

## Introduction

Understanding the evolution of adaptive plasticity has been the focus of a rich and growing literature (Dewitt and Scheiner 2004). Despite its seeming superiority over a fixed phenotype, adaptive plasticity of continuous traits is less ubiquitous than might be naively expected. At best, adaptive trait plasticity can account for only some of the wide ecological range of many species, and instead genetic differentiation and local adaptation are commonly observed (Hereford 2009). Recently (Scheiner and Holt 2012), through the use of simulation models, we showed that environmental variation and uncertainty affect whether or not trait plasticity is favored over local adaptation. (We urge reading that paper prior to this current missive as the results presented here build on those.) Different sources of variation experienced by individuals and lineages – arising from the amount and timing of dispersal, from temporal variation, and even from the genetic architecture underlying the phenotype – have contrasting, interacting, and at times unexpected effects. Running through all of those simulations and earlier theoretical work (e.g., Moran 1992; Gavrilets and Scheiner 1993) is the importance of dispersal rate. In a spatially heterogeneous environment, the probability of moving to a new environment greatly influences the environmental variation and uncertainly experienced by an organism and a genetic lineage, and thus how selection is expected to act on plasticity. (The theory of plasticity evolution has an extensive history, reviews of which can be found in Pigliucci (2001), Berrigan and Scheiner (2004), and Scheiner and Holt (2012)).

Until now in analyses of the evolution of dispersal, dispersal rate has been treated as a fixed (albeit probabilistic) characteristic of a population. Yet we know that the propensity to disperse can be genetically variable and subject to natural selection, and therefore dispersal itself can evolve (e.g., Roff 1990; Cody and Overton 1996; Donohue et al. 2005; Llewelyn et al. 2010). For reviews of the extensive literature on the evolution of dispersal see Clobert et al. (2001), Bullock et al. (2002), and Ronce (2007).

The evolution of dispersal raises the question of what happens when the propensity to disperse and trait plasticity both have genetic variation and are allowed to evolve jointly. Even in the absence of plasticity, in a spatially distributed population in a uniform environment there is always some positive selection on dispersal rate due to local kin competition (Frank 1986), sometimes referred to as the Hamilton–May effect (Hamilton and May 1977). When environmental heterogeneity is added, maladaptation of dispersing individuals who move away from local environments to which they are adapted acts as a countervailing force that selects against dispersal. This effect can be frequency-dependent, though, because very high dispersal rates indirectly decrease the overall amount of local adaptation maintained in a spatially heterogeneous environment and, thus, this component of selection against dispersal (Billiard and Lenormand 2005). With regard to the evolution of plasticity, previous theoretical work has shown that low dispersal rates tend to favor genetic differentiation over adaptive plasticity, with the reverse outcome arising for high dispersal rates (e.g., Moran 1992; Gavrilets and Scheiner 1993; Scheiner and Holt 2012). Is one of those two states favored when both characteristics can evolve?

In this paper, we show that when dispersal rate itself evolves, those tendencies towards either one or the other extreme (genetic differentiation with little plasticity, or adaptive plasticity and scant local adaptation) are reinforced. The results are strongly dichotomous outcomes that depend on starting conditions and other factors, such as, the stage of the life cycle experiencing selection, the cost of plasticity, and linkage between genes for trait plasticity and dispersal. Our previous simulation study (Scheiner and Holt 2012) found that mixed outcomes (partial differentiation and partial plasticity) were possible for some parameter combinations. Those intermediate outcomes tend to disappear, we now find, when both traits can evolve.

## Model Structure

### Overview

The model was an individual-based simulation (summary of parameters in Table 1) using a gene-based model of adaptation to an environmental gradient. It assumed that the optimal phenotype changes in a linear fashion along that gradient, and the phenotypes of individuals can be expressed by a linear reaction norm. The genes have expression that are either responsive to the environment (plastic loci) or not (nonplastic loci). Adaptation can occur by two routes: genetic differentiation in which the allelic values of the plastic loci go to zero (i.e., are not expressed), or phenotypic plasticity in which the allelic values of the nonplastic loci go to zero. Because the optimal phenotype changes in a linear fashion along the gradient, and the environmental responsiveness of the plastic loci is linear, the plasticity optimum (where the realized trait in each habitat is the local optimum) is a possible outcome. Although we present these outcomes as a dichotomy, intermediate outcomes are possible in which individuals express the optimal phenotype in a particular environment through nonzero values of both the plastic and nonplastic loci. See figure 3 in Scheiner (1998) for a visualization of such an intermediate outcome. Our previous paper in this journal (Scheiner and Holt 2012) explored in detail the conditions that favor plasticity versus genetic differentiation outcomes when dispersal rate is a fixed trait.

**Table 1:** Summary of the model parameters

Fixed parameters
Number of nonplastic, plastic, and dispersal loci = 1 each
Length of the environmental gradient = 50 demes
Steepness of the gradient (change in optimum in adjacent demes) = 0.4 units (except as noted)
Strength of selection within demes (σ) = 2 units
Population size = 100 individuals/deme
Number of generations = 20,000
Parameters explored
Cost of plasticity
Evolution of dispersal rate: no or yes
Linkage: none or complete
Initial dispersal rate
Initial mean reaction norm
Life-history pattern: selection before dispersal versus dispersal before selection

This model formulation is different from most models of plasticity evolution (Berrigan and Scheiner 2004). Optimality models and quantitative genetic models both take one of two forms: a reaction-norm model where the focus is on the parameters of the reaction norm (e.g., the elevation and the slope of a linear reaction norm), or a character-state model where trait expression in each environment is treated as a separate trait (De Jong 1995). Genic models are neither, with trait values in a given environment and reaction norm parameters both arising as epiphenomena of gene expression.

### Specifics

The model was implemented in Fortran 77 (computer code is given in Appendix S2). The metapopulation consisted of a linear array of 50 demes. An environmental gradient was created by varying the optimal value of a single trait (phenotype) in a linear fashion along the array from −9.8 to +9.8 arbitrary units at the ends of the gradient, that is, the optimal phenotype in adjacent demes differed by 0.4 units. An individual's phenotype (trait value) was determined by two diploid loci: one plastic locus and one nonplastic locus. The loci contributed additively to the trait. Allelic values at the plastic locus were multiplied by an environment-dependent quantity before summing all allelic values. The effect of the environment (*E*_*i*_ for deme *i*) on the phenotypic contribution of each unit plastic allelic value varied in a linear fashion, with a slope of 0.04 units [*E*_*i*_ = 0.04 (*i*−25.5)]. The phenotype of each individual was determined at the time of development, and is given by:


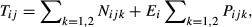
(1)

where *T*_*ij*_ is the phenotype of the *j*th individual that develops in the *i*th environment (deme), *N*_*ijk*_ is the allelic value of the *k*th nonplastic allele of that individual, and *P*_*ijk*_ is the allelic value of the *k*th plastic allele. In our model there was no random component of phenotypic variation. Because both the environmental gradient and the effect of plasticity alleles on the phenotype were linear, perfect adaptation through either genetic differentiation or plasticity was possible. For a given genotype, Σ*N*_*ijk*_ can also be thought of as the intercept of its reaction norm, or the phenotype of the individual in the absence of plasticity, and [slope(*E*_*i*_)Σ*P*_*ijk*_] can be thought of as the slope of its reaction norm.

Life history events occurred in one of two sequences: (1) birth, followed by development (i.e., the phase in the life cycle when the phenotype is determined), then dispersal, selection, and reproduction (which we call “move first”); or alternatively, (2) birth, development, selection, dispersal, and then reproduction (which we denote by “select first”). Selection was based on survival with the probability of surviving being a Gaussian function of the difference between an individual's phenotype and the locally optimal phenotype. Fitness (the probability of surviving) was determined as:


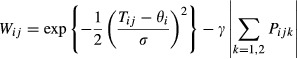
(2)

where *W*_*ij*_ is the fitness of the *j*th individual undergoing selection in the *i*th environment, *T*_*ij*_ is the phenotype of that individual, *θ*_*i*_ is the optimal phenotype in that environment, *σ* is the strength of selection (selection weakens as *σ* increases), and the second term on the right is the cost of plasticity, where *γ* is the per-unit cost and the rest of the term is the magnitude of the plasticity of the trait as defined above. When there was a cost (*γ* > 0), plastic alleles created a linear decrease in fitness regardless of the expression of those alleles in a given environment. This is equivalent, for example, to a cost of maintaining the machinery for the expression of trait plasticity. Costs were scaled to indicate the percentage decrease in fitness for individuals that expressed the optimum reaction norm, that is, the reaction norm matching the slope of *θ*.

**Figure 1:**
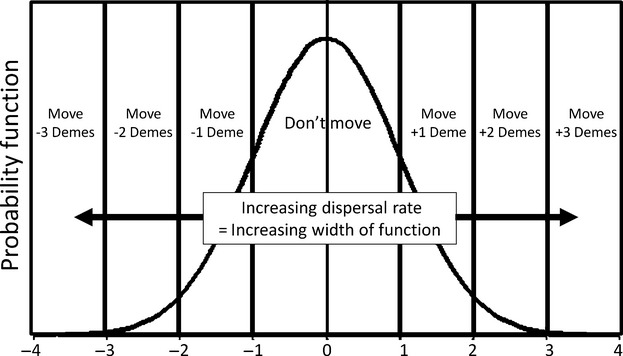
Probability density function for the random variable determining the likelihood that an individual would move between demes and the distance of that movement. Shown is a dispersal probability of 32%. An increase or decrease in that probability is equivalent to increasing or decreasing the width of the function.

The dispersal probability and the distance moved were determined by one diploid locus: 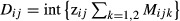, where *D*_*ij*_ gives the distance and direction moved of the *j*th individual that develops in the *i*th environment (deme), *M*_*ijk*_ is the allelic value of the *k*th dispersal allele of that individual, *z*_*ij*_ is a sequence of zero-mean, unit-variance Gaussian random deviates chosen independently for each individual, and *int* is the integer function (truncation of the decimal portion of the number). The magnitude of *D*_*ij*_ determined the number of demes moved, with the sign determining the direction of movement. The probability of moving and the average distance moved were correlated (Fig. 1). The term in brackets is a zero-mean Gaussian with standard deviation (SD) equal to the magnitude of the sum of the dispersal alleles. If it was between −1 and 1 (the Gaussian was within 1 SD of its mean), *D*_*ij*_ = 0 and the individual did not move. For example, if the sum of dispersal alleles was 1, the Gaussian had unit variance, and the probability of dispersal was 32%, because 68% did not disperse at all (the probability that a unit-variance Gaussian is within 1 SD of the mean). The fraction of individuals that instead dispersed 1 deme (half in either direction) was 27% (the probability that a unit-variance Gaussian has a magnitude between 1 and 2); 5% dispersed 2 demes (the probability that a unit-variance Gaussian has a magnitude between 2 and 3), and so forth. An increase in the sum of dispersal alleles gives an increase in the variance of the Gaussian in brackets and increased the dispersal probability and the average distance moved. Individuals that would otherwise disperse beyond the end of the gradient moved to the terminal demes. Dispersal per se had no cost; survival during dispersal was 100%.

Reproduction was accomplished by assembling pairs of individuals within a deme at random with replacement, with each pair producing one offspring, then repeating until the carrying capacity of that deme was reached (in our simulations, this was 100 individuals per deme). This procedure assumes soft selection, in that local population size was determined independently of the outcome of selection. It also assumes that the spatial scale of reproduction and mating matches that of density dependence and the grain of the selective environment. (We will relax the assumption of soft selection in a future contribution.)

Each simulation was initialized with 100 individuals being born in each deme. For each individual in the initial generation, all allelic values at each locus were identical (i.e., there was no genetic variation). For most simulations, initial values for the nonplastic and plastic alleles were set to 0. For some simulations where we explored the effects of starting conditions, the values of the plastic alleles differed from 0 i.e., a non-zero reaction norm), but again with no initial genetic variation. Similarly, we varied the initial dispersal rate of the metapopulation, but set it initially to be genetically uniform.

**Figure 2:**
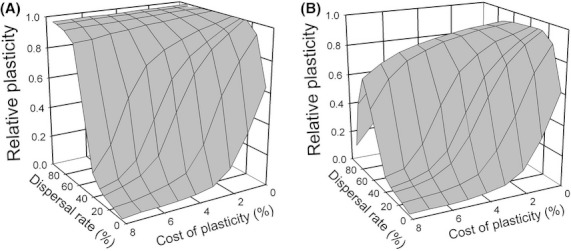
The interaction of dispersal rate and cost of plasticity on the evolution of phenotypic plasticity, where the cost is scaled as the percentage decrease in fitness for an individual expressing the optimal reaction norm. In these simulations dispersal rate was a fixed trait and loci were unlinked. (A) Selection before dispersal (select first). (B) Dispersal before selection (move first). A value of 1.0 for relative plasticity indicates a pure plasticity outcome.

When new offspring were generated, each allele mutated with a probability of 10%. Lower mutation rates mainly changed the time-scale over which evolution occurs, rather than the eventual outcome, so we used a high mutation rate to speed up the simulations. (The effects of mutation rate on model outcome are shown in figure 2 of (Scheiner and Holt 2012).) When a mutation occurred, the allelic value was changed by adding a Gaussian deviate (mean of 0 and a SD of 0.1 units) to the previous allelic value (i.e., this is an infinite-alleles model). All simulations were run for 20,000 generations to ensure that the equilibrium point (the point after which all calculated quantities showed no further directional trend) was reached (Scheiner and Holt 2012). Each parameter combination was replicated 20 times and the results shown are the means of those replicates. Coefficients of variation of reported parameters were generally low (5–20%), but could be substantially higher in regions with abrupt transitions.

The reaction norm is a mathematical function describing how the phenotypic expression of a given genotype varies among environments. The plasticity of a linear reaction norm is best described by the slope of the function. In our model, the slope of the reaction norm is the product of the slope of *E*_*i*_ and the sum of the values of the plasticity alleles (i.e., the right-hand sum in eq. 1). For these simulations, as the slope of *E*_*i*_ was identical, the final outcome was measured as the average across all demes of the sum of the values of the plasticity alleles for each individual. That is, 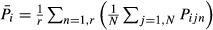, where 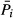 is the mean plasticity of the *i*th deme over all *r* runs, *N* = 100 is the number of individuals per deme, and *P*_*ijn*_ is the sum of the values of the plasticity alleles of the *j*th individual developing in the *i*th deme in the *n*th run. The overall mean plasticity 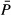 is the average of 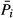 across demes, and is given by 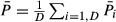, where *D* is the number of demes. (The order of averaging, over runs within demes first or over demes within runs first, does not affect the final average, because the number of demes is the same for all runs. Mean plasticity was calculated at each generation.) The average plasticity was standardized to the optimal reaction norm (giving the relative plasticity) so that a pure plasticity outcome would have a value of 1 and a pure differentiation outcome would have a value of 0. Values outside this range were possible; that is, it was possible to achieve a reaction norm with a slope steeper than the optimal value (>1) or in a direction opposite from the optimal value (<0).

## Results

### Baseline – fixed dispersal and costs of plasticity

We begin by establishing baseline conditions observed with a non-evolving dispersal rate. As we showed previously (Scheiner and Holt 2012), dispersal rate interacts with life history pattern. When there is no cost of plasticity and selection occurs prior to dispersal (select first), so that development and selection happen in the same environment, moderate to high dispersal rates (15% and above) strongly favor plasticity (right edge in Fig. 2A). In contrast, selection after dispersal (move first) means that development and selection can occur in different environments, favoring plasticity most at intermediate rates of dispersal (20–60%; right edge in Fig. 2B). At all dispersal rates, plasticity is less favored in the move-first life history pattern, and dispersal rates above 50% begin to further disfavor plasticity for the move-first scenario, because dispersal prior to selection adds uncertainty to the environment of selection, especially at very high dispersal rates.

Adding a cost to trait plasticity naturally disfavors adaptive plasticity and as costs increase this pushes the system first to an intermediate state, then to pure genetic differentiation. This occurs for both life history patterns and at all dispersal rates. For the select-first life history pattern at high dispersal rates, plasticity is still quite high; at higher costs, it is likely that plasticity would be disfavored as well. Therefore, if plasticity is sufficiently costly it is unlikely to evolve. In our simulations, even a small fitness decrement of 4% for individuals expressing the optimal plasticity was sufficient to disfavor plasticity at low dispersal rates.

### Dispersal rate evolution

Before we can understand the joint evolution of trait plasticity and dispersal rate, we must establish the baseline, equilibrium rate to which dispersal will evolve, were plasticity absent or fixed. Because of the Hamilton–May effect, dispersal rate does not evolve to zero even in a uniform environment. If the population is substructured into demes so that an individual is more likely to compete with kin than non-kin, selection favors some dispersal. We varied the amount of environmental heterogeneity, and consequentially the strength of selection on dispersal, by altering the slope of the gradient of optimal phenotypes. (The within-deme strength of stabilizing selection was not varied.) A slope of zero indicates a uniform environment with stabilizing selection for the same optimum along the entire gradient of demes.

**Figure 3:**
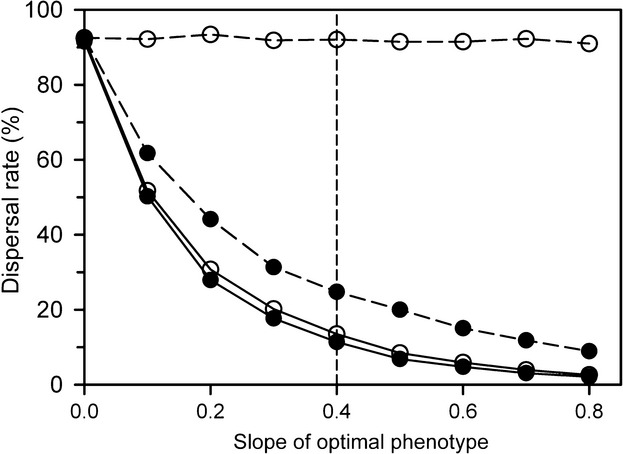
The evolution of dispersal rate with varying amounts of environmental heterogeneity (the slope of the gradient of optimal phenotypes) when only genetic differentiation is possible (no plastic loci, solid symbols) or when only plasticity is possible (no non-plastic loci, open symbols). Solid lines indicate dispersal before selection (move first); dashed lines indicate selection before dispersal (select first). For these simulations, there was no cost of plasticity. The vertical dashed line indicates the slope used in the other simulations in this paper.

First we examined dispersal rate evolution when the only type of adaptation was through genetic differentiation by setting the number of plastic loci to zero (Fig. 3, *solid symbols*). In a uniform environment, dispersal rate evolved to ca. 92% for both life history patterns. As the environment along the gradient became more varied, the equilibrium dispersal rate declined, with the rate of decline greater for the move-first life history pattern. Environmental heterogeneity selects against dispersal because individuals that disperse are no longer in their optimal environment. For the environmental heterogeneity used in the rest of the simulations, the equilibrium dispersal rate was about 25% for the select-first life history pattern and about 11% for the move-first life history pattern (Fig. 3).

If the only type of adaptation was through plasticity, for the select-first life history pattern there was no decline in the final dispersal rate with an increase in the slope of the selection gradient, with final dispersal remaining above 90% (Fig. 3, *open symbols, dashed line*). This is because for this life history pattern, perfect adaptation through plasticity is possible for any linear gradient. For the move-first life history pattern, however, the final dispersal rate dropped with an increasing slope, because individuals that move undergo selection in a different environment from development, so there is a fitness penalty for dispersal (Fig. 3, *open symbols, solid line*).

### Joint evolution

**Figure 4:**
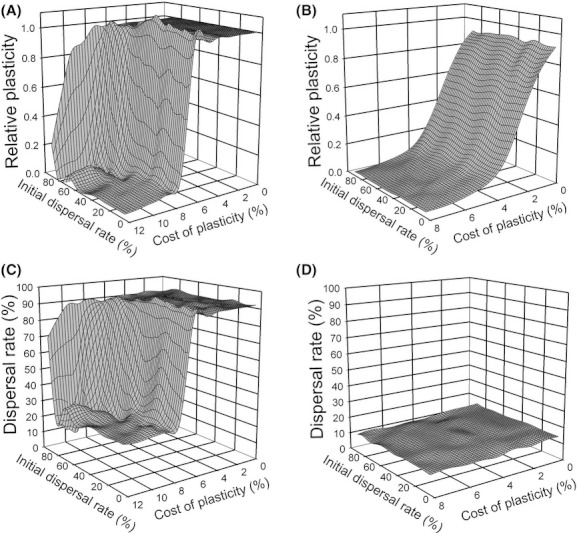
The interaction of dispersal rate and cost of plasticity on the joint evolution of phenotypic plasticity and dispersal rate. Cost is scaled as the percentage decrease in fitness for an individual expressing the optimal reaction norm. In these simulations loci were unlinked. (A) Evolution of plasticity with selection before dispersal (select first). (B) Evolution of plasticity with dispersal before selection (move first). (C) Evolution of dispersal rate with selection before dispersal (select first). (D) Evolution of dispersal rate with dispersal before selection (move first).

Allowing dispersal rate to jointly evolve with trait plasticity can greatly change the outcome, although this result depends on the life history pattern. When selection occurs prior to dispersal (select first), the outcome is nearly dichotomous, either pure plasticity or pure genetic differentiation (Fig. 4A). Genetic differentiation is favored when plasticity costs are high at all dispersal rates, although at the highest initial dispersal rate there is still some plasticity even at the highest cost. The propensity to disperse evolves so as to mirror the pattern of trait plasticity (Fig. 4C). When trait plasticity is high, the final dispersal rate is also high (ca. 90%); when genetic differentiation is favored, the system evolves to moderate dispersal rates (ca. 25%). These dispersal rates are similar, respectively, to those when only genetic differentiation or plasticity are possible (Fig. 3, vertical dashed line). For high costs and the highest initial dispersal rates, the final dispersal rate is intermediate.

When selection occurs after dispersal (move first), in contrast, the final outcome is determined entirely by the cost of plasticity and the effects of high initial dispersal rate are now gone (Fig. 4B). The propensity to disperse evolves to a low rate (ca. 10%) for all parameter combinations (Fig. 4D). High dispersal rates create uncertainty when dispersal happens before selection. When dispersal can evolve, the system evolves in effect to reduce uncertainty. This uncertainty effect does not exist when selection occurs before dispersal, because the environment at selection is the same as that at development. These outcomes are similar to those when only genetic differentiation or plasticity are possible (Fig. 3, vertical dashed line).

### Effects of linkage

If trait plasticity and dispersal rate can affect each other's evolution, genetic linkage might alter that dynamic. Because there are three loci in this system, we first need to establish what happens with linkage and fixed dispersal rates. Linkage between the plastic and nonplastic loci does not affect the evolutionary equilibrium (see Appendix Fig. S1, compare with Fig. 2) except at very low dispersal rates and zero cost of plasticity, where linkage favors plasticity. The transient dynamics (data not shown) show brief periods of movement away from the overall trend or from equilibrium as new mutations enter the system, but the high mutation rate used in our simulations erodes any linkage disequilibrium within tens of generations. These effects might differ at lower mutation rates. In all of the results presented in this paper we used only no linkage or complete linkage. We examined the effects of partial linkage (recombination rates of 12.5%, 25% and 37.5%) and found results identical to those with complete linkage (results not shown).

**Figure 5:**
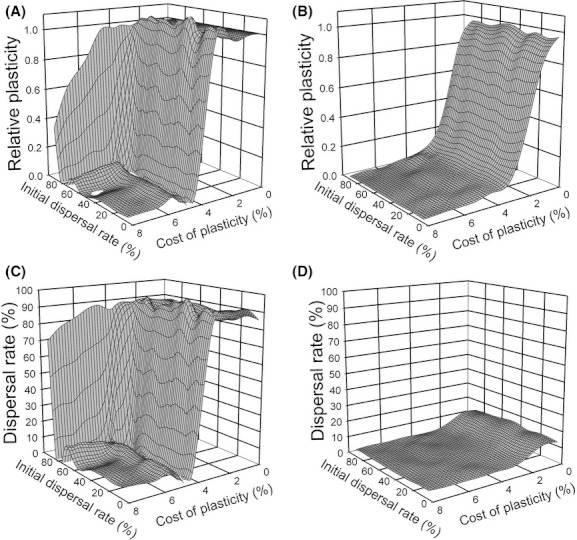
The effects of complete genetic linkage on the interaction of dispersal rate and cost of plasticity on the joint evolution of phenotypic plasticity and dispersal rate. (A) Evolution of plasticity with selection before dispersal (select first). (B) Evolution of plasticity with dispersal before selection (move first). (C) Evolution of dispersal rate with selection before dispersal (select first). (D) Evolution of dispersal rate with dispersal before selection (move first).

By contrast, when dispersal can evolve and all three types of loci are linked, the joint evolutionary dynamic of plasticity and dispersal rate are altered to some degree. For both life history patterns, the system is more likely to move towards genetic differentiation, accentuating the effects of the cost of plasticity (Fig. 5, compare with Fig. 4). For the select-first scenario, these effects occur everywhere except at low costs or high initial dispersal rates. For the move-first scenario, adaptive plasticity is favored only at very low costs of plasticity. Overall, for both life history patterns the threshold between a pure plasticity outcome and a pure genetic differentiation outcome is even sharper, making the ultimate outcomes even more dichotomous. With linkage, the pure genetic differentiation outcomes also push the final dispersal rates even lower, close to zero, for both life history patterns. The result is either a system with no plasticity and extremely low dispersal rates, or one with optimal plasticity and high dispersal rates, with few systems in between.

### Effects of starting conditions

The initial dispersal rate clearly affects the final state of the system (Figs. 4, 5), at least for the select-first scenario. We also explored whether the same was true for the initial amount of trait plasticity by simultaneously varying the initial dispersal rate and the initial relative plasticity. An initial relative plasticity value of 1 means that all individuals in the metapopulation expressed the optimal reaction norm at generation 1. A value above 1 means that all individuals had a reaction norm that was steeper than the optimum, and a value of 0 means that all individuals had no plasticity (and also no differentiation).

**Figure 6:**
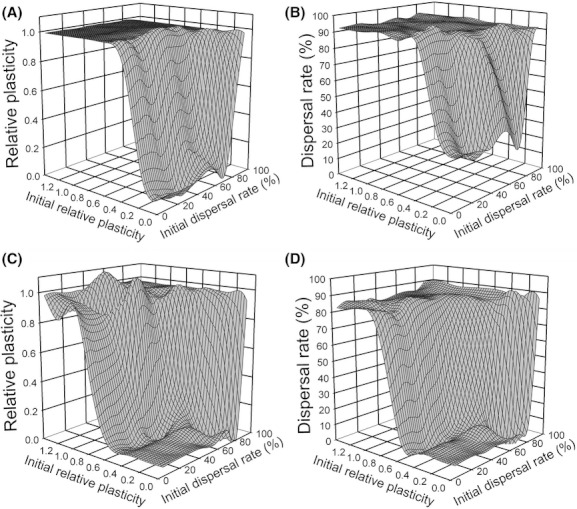
The effects of starting conditions and genetic linkage on the interaction of initial dispersal rate and initial relative plasticity. In these simulations selection occurred before dispersal (select first). (A) Relative plasticity with no genetic linkage, the cost of optimal plasticity was 8%. (B) Dispersal rate at equilibrium with no genetic linkage, the cost of optimal plasticity was 8%. (C) Relative plasticity with genetic linkage, the cost of optimal plasticity was 5%. (D) Dispersal rate at equilibrium with genetic linkage, the cost of optimal plasticity was 5%.

We first examined the select-first life history scenario. For these simulations the costs of optimal plasticity were set at values that resulted in a range of equilibrium values as the initial dispersal rate was varied (8% for unlinked loci, Fig. 4A; 5% for linked loci, Fig. 5A). For both unlinked loci and linked loci, both high initial dispersal rates and high initial relative plasticity resulted in a system with near optimal plasticity and high dispersal rates (Fig. 6).

**Figure 7:**
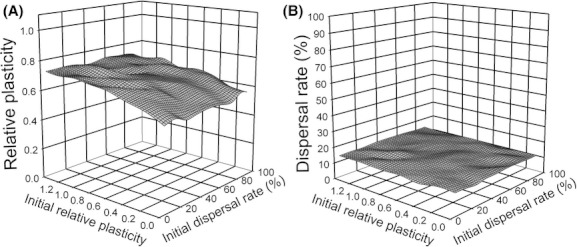
The effects of starting conditions on the interaction of initial dispersal rate and initial relative plasticity. In these simulations dispersal occurred before selection (move first), the cost of optimal plasticity was 2%, and loci were unlinked. (A) Relative plasticity. (B) Dispersal rate at equilibrium.

The move-first life history scenario resulted in a markedly different behavior. For these simulations the cost of optimal plasticity was set to 2%, because this was a value that gave intermediate outcomes (Fig. 4B). In this instance, for unlinked loci the initial relative plasticity had a modest, if noticeable, effect on the final relative plasticity, but no effect on the final dispersal rate (Fig. 7). The initial dispersal rate had no effect on either. If the loci were linked, consistent with the previous results (Fig. 5B), absolute values of relative plasticity and dispersal rate were lower, but the overall pattern of response was the same (results not shown).

### Transient dynamics

**Figure 8:**
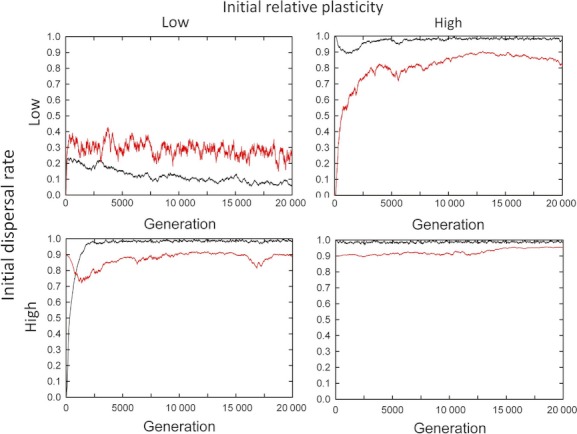
Examples of the transient behavior of plasticity (black) and dispersal rate (red) for different values of initial relative plasticity and dispersal rate without linkage. In these simulations selection occurred before dispersal (select first) and the cost of optimal plasticity was 8%.

**Figure 9:**
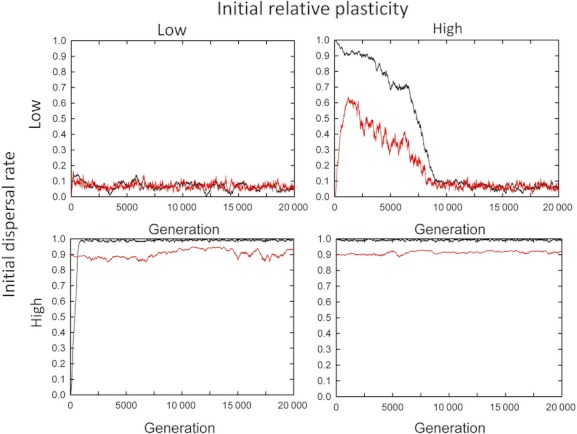
Examples of the transient behavior of the plasticity (black) and dispersal rate (red) for different values of initial relative plasticity and dispersal rate with linkage. In these simulations selection occurred before dispersal (select first) and the cost of optimal plasticity was 5%.

We next turn to the question of whether dispersal rate tends to drive the evolution of plasticity, or the reverse. We addressed this question by examining the transient behavior of the system under different initial conditions. For the select-first life history scenario when the loci were unlinked, if either trait was initially low, and the other high, the low trait rapidly increased (upper right and lower left panels in Fig. 8). The other trait initially declined somewhat, but increased again once the other trait reached high levels. High plasticity coupled with high dispersal remained that way. If the two traits both start low, they initially jumped to moderate values, but then equally quickly started to drift lower (upper left panel of Fig. 8). The behavior of the system is similar if the loci were linked (Fig. 9), except when the system started with high plasticity and a low dispersal rate. In this instance, trait plasticity declined swiftly enough that dispersal rate rose and then declined again. In this particular run, at about 7000 generations dispersal rate had declined sufficiently to precipitate a rapid decline in trait plasticity. So the ultimate outcome was the result of the combined dynamics of both traits. For both unlinked and linked loci, if both dispersal and plasticity started low, they remained low, and if both were initially high, they remained high.

For the move-first life history scenario (see Appendix Fig. S2), dispersal evolution stayed low or quickly declined if it began at a high value. In contrast, trait plasticity more gradually evolved to an intermediate level. So, dispersal rate evolution is being driven by other processes and trait plasticity is responding to dispersal evolution. This is consistent with the results in Figure 7, where the final dispersal rate was independent of the initial values of either dispersal rate or trait plasticity.

## Discussion

In a spatially heterogeneous environment, dispersal rate is key to whether trait plasticity is favored. A higher dispersal rate increases the environmental variability experienced by a lineage; conversely, a low dispersal rate reduces that variability. We found that letting dispersal rate also evolve enhances these effects so that outcomes tend to be more dichotomous. Systems tended to either high plasticity coupled with high dispersal, or low plasticity and low dispersal. We had expected this result and also that, perhaps, one of the two outcomes would generally prevail. What we did not expect was the extent to which the outcome depended on starting conditions, in particular on whether the system started at high values for one or the other trait. Adding a cost of plasticity tended to drive the system to genetic differentiation, although this effect also depended on initial conditions. Genetic linkage between trait plasticity loci and dispersal loci further enhanced this strong dichotomy. And, all of these effects depended on organismal life history pattern: as expected, whether selection occurred before or after dispersal has a strong effect on the joint evolution of dispersal and plasticity. In our previous paper (Scheiner and Holt 2012), we concluded that patterns of environmental variation can interact with life history patterns and genetic architecture in unexpected ways. We reinforce that general observation with the current results.

### What drives plasticity evolution?

We found that for the select-first life history scenario, if the system starts at the optimal reaction norm, then dispersal rate can be driven to high values (Figs. 6B, 8). Each trait responds to the other, and dispersal rate can rise fast enough to cross into a new basin of attraction, with the boundaries of those basins changing when linkage is added (Fig. 6D, 9). This codependence is similar to the effect of local adaptation on the evolution of dispersal where weakened local adaptation due to high dispersal reinforces the evolution of dispersal (Ronce 2007). In our case, though, the weakening of local adaptation comes about through high plasticity. These effects are absent for the move-first life history scenario (Figs. 7B, A2); for that life history pattern, the optimal reaction norm is never the equilibrium solution under the conditions explored in these simulations (Fig. 2B). For the move-first life history scenario, the cost of plasticity was the primary determinant of the outcome.

These results might help explain why adaptive plasticity is less common than we might naively expect. Many processes can favor local mating, selfing or philopatry. These processes can be driven by factors other than trait plasticity, such as selection for local adaptation of other, nonplastic traits. This selection for lower dispersal rates might indirectly push systems to low plasticity for all traits. In our model dispersal, per se, had no fitness cost. Previous models show that adding a cost to dispersal lowers dispersal rates (e.g., Roff 1975; Hamilton and May 1977; Comins et al. 1980; Murrell et al. 2002). Such costs of dispersal are, thus, most likely to further disfavor plasticity.

In our previous paper, where we assumed that dispersal rates were fixed and there was no cost for plasticity (Scheiner and Holt 2012), we found that for the select-first life history pattern, the optimal reaction norm was the likely outcome under nearly all circumstances except at very low dispersal rates. That result coincides with the current results of optimal reaction norms when plasticity was favored. In contrast, in our previous results for the move-first life history pattern, the outcome ranged from partial plasticity to hyperplasticity (a reaction norm substantially steeper than the optimum) depending on the genetic architecture (number of loci) and the pattern and amount of the temporal variation in phenotypic optima. That result differed from the current results where plasticity was disfavored under nearly all conditions explored. We have not examined the effects of temporal variation on plasticity evolution when dispersal rate can also evolve. In our previous simulations, the effects of temporal variation depended on the dispersal rate, so it remains to be seen whether adding such variation when dispersal rates can also evolve might favor plasticity for the move-first life history scenario.

### Predictions

Our model makes several predictions concerning patterns of linkage within genomes and patterns of trait plasticity among populations. Regarding linkage, loci affecting traits that have high plasticity should generally be unlinked to loci that affect dispersal rates, unless the cost of plasticity is low. Conversely, when loci affecting those traits are linked to dispersal loci, we predict a dichotomous pattern of plasticity among populations. Populations should have either high plasticity and dispersal rates, or low plasticity and dispersal rates. When loci are unlinked, variation among populations is more likely to be continuous and to have intermediate levels of plasticity. Regarding geographic patterns, if dispersal rates vary among populations, there should be an abrupt transition between regions that show genetic differentiation among populations and those that show high levels of trait plasticity. We are unaware of any data that provide a test of these hypotheses, although such tests could conceivably be made in a variety of systems, including the cricket *Allonemobius socius* (Mousseau and Roff 1989, 1995; Winterhalter and Mousseau 2007), the snail *Physa heterostropha* (Dewitt 1998; Dewitt et al. 1999; Langerhans and Dewitt 2002), and the plant *Impatiens capensis* (Schmitt 1993; Dudley and Schmitt 1995; Donohue and Schmitt 1999; Donohue et al. 2000).

### Costs of plasticity

In our simulations, even relatively low costs of plasticity disfavored trait plasticity, especially for the move-first life history pattern. Searches for costs of plasticity usually have failed to find them, suggesting that they are small or absent most of the time (e.g., Scheiner and Berrigan 1998; van Kleunen et al. 2000; Weinig et al. 2006; Steiner and Buskirk 2008; Aubret and Shine 2010). However, such searches were done for traits known to have substantial plasticity – exactly where one would predict costs to be low, because low costs make it more likely plasticity will evolve. We need to measure such costs for traits that have low levels of plasticity. Admittedly such experiments are hard because of the low power of any test. One solution is to introduce alleles for high plasticity into populations with low plasticity to see if costs emerge. The other solution is to look for costs specifically in populations with high dispersal rates and a select-first life history pattern, the conditions that our model predicts should favor plasticity even when costs are high.

In our simulations, the cost of plasticity was proportional to the absolute value of the sum of the plasticity alleles (eq. 2). That is, cost was a function of the net total allelic effects. Alternatively, the cost could be modeled as proportional to the sum of the absolute values of each plasticity allele, so that the cost would be a function of each allele. These differences reflect possible biological differences in how costs are manifest. Costs that are due to the total effect across alleles might occur at the whole phenotype level. An example would be the cost of maintaining additional sensory equipment. In contrast, costs that are due to the plasticity of individual alleles might occur at the genetic level. An example would be the cost of maintaining machinery to vary gene expression levels. This latter form of costs is likely to further disfavor plasticity as such costs would tend to select against the maintenance of genetic variation among plastic alleles. Current measures of the cost of plasticity focus on the total effect on fitness; by contrast, little is known about costs of plasticity at the level of specific alleles. For those instances where costs have been found (e.g., Poulton and Winn 2002; Lind and Johansson 2009; Maherali et al. 2010), information is lacking on the exact mechanisms underlying those costs.

### For future work

The simulations we report here have only scratched the surface of this topic. We close by sketching avenues for future exploration. As has already been mentioned, we know that temporal variation can interact with dispersal rate. Also, we have yet to examine the effects of hard selection for either fixed or evolving dispersal rates. We previously showed (Scheiner and Holt 2012) that the number of loci has an effect on whether plasticity is favored for the move-first life history pattern. In the current simulations, we were interested in the effects of linkage and so modeled only one locus of each type. Including more loci would allow for more complex patterns of genetic architecture. For example, the exact order of loci on a chromosome matters if linkage is partial. We could also explore the evolution of linkage by having complete linkage of two of the loci while making them both unlinked to the third locus (e.g., the effect of being on different chromosomes). It is not obvious if such complexities would change the overall patterns found here. Patterns of genetic architecture are some of the easiest to measure in these days of increasingly cheap gene sequencing, providing opportunities for empirical tests that are lacking for most other predictions of models of the evolution of plasticity. All of these themes will be the subject of future papers.

Our results point to the importance of looking beyond a single trait and its plasticity to understand trait evolution. We need to consider the entire life history of an organism (e.g., when the phenotype is determined relative to when selection and movement occur) and the potential for that life history to evolve. Dispersal itself can be context dependent (i.e., be a plastic behavior), adding yet another level of complexity. As models attempt to embrace these additional complexities, it behooves us to attempt to link such models to specific systems. Simple models such as ours are useful for their generality. They make qualitative predictions that are likely to hold across many taxa. Complex models tailored to specific systems are more realistic, but are also inherently less general. Grounding our general models in more specific models that match the attributes of particular species should permit the specification of parameter values and the refinement of empirical predications, beyond the qualitative conclusions we have here drawn.

## Supporting Information

**Appendix S1**. Figure S1. The effects of complete genetic linkage on the interaction of dispersal rate and cost of plasticity on the evolution of phenotypic plasticity. In these simulations dispersal rate was a fixed trait. (A) Selection before dispersal (select first). (B) Dispersal before selection (move first). Figure S2. Examples of the transient behavior of plasticity (black) and dispersal rate (red) for different values of initial relative plasticity and dispersal rate without linkage. In these simulations dispersal occurred before selection (move first) and the cost of optimal plasticity was 2%.

**Appendix S2**. Computer code used for the simulations (copyright 2012 Samuel M. Scheiner). This program is free software: you can redistribute it and/or modify it under the terms of the GNU General Public License as published by the Free Software Foundation, either version 3 of the License, or (at your option) any later version. This program is distributed in the hope that it will be useful, but WITHOUT ANY WARRANTY; without even the implied warranty of MERCHANTABILITY or FITNESS FOR A PARTICULAR PURPOSE. See the GNU General Public License for more details <http://www.gnu.org/licenses/>.
