# Supplementary material for: The genetics of phenotypic plasticity. XI. Joint evolution of plasticity and dispersal rate
Source: Ecol Evol. 2012 Jul 20;2(8):2027–39. doi: 10.1002/ece3.327 (PMC3434005; doi:10.1002/ece3.327)

Appendix A

Figure A1. The effects of complete genetic linkage on the interaction of dispersal rate and cost of plasticity on the evolution of phenotypic plasticity. In these simulations dispersal rate was a fixed trait. (A) Selection before dispersal (select first). (B) Dispersal before selection (move first).


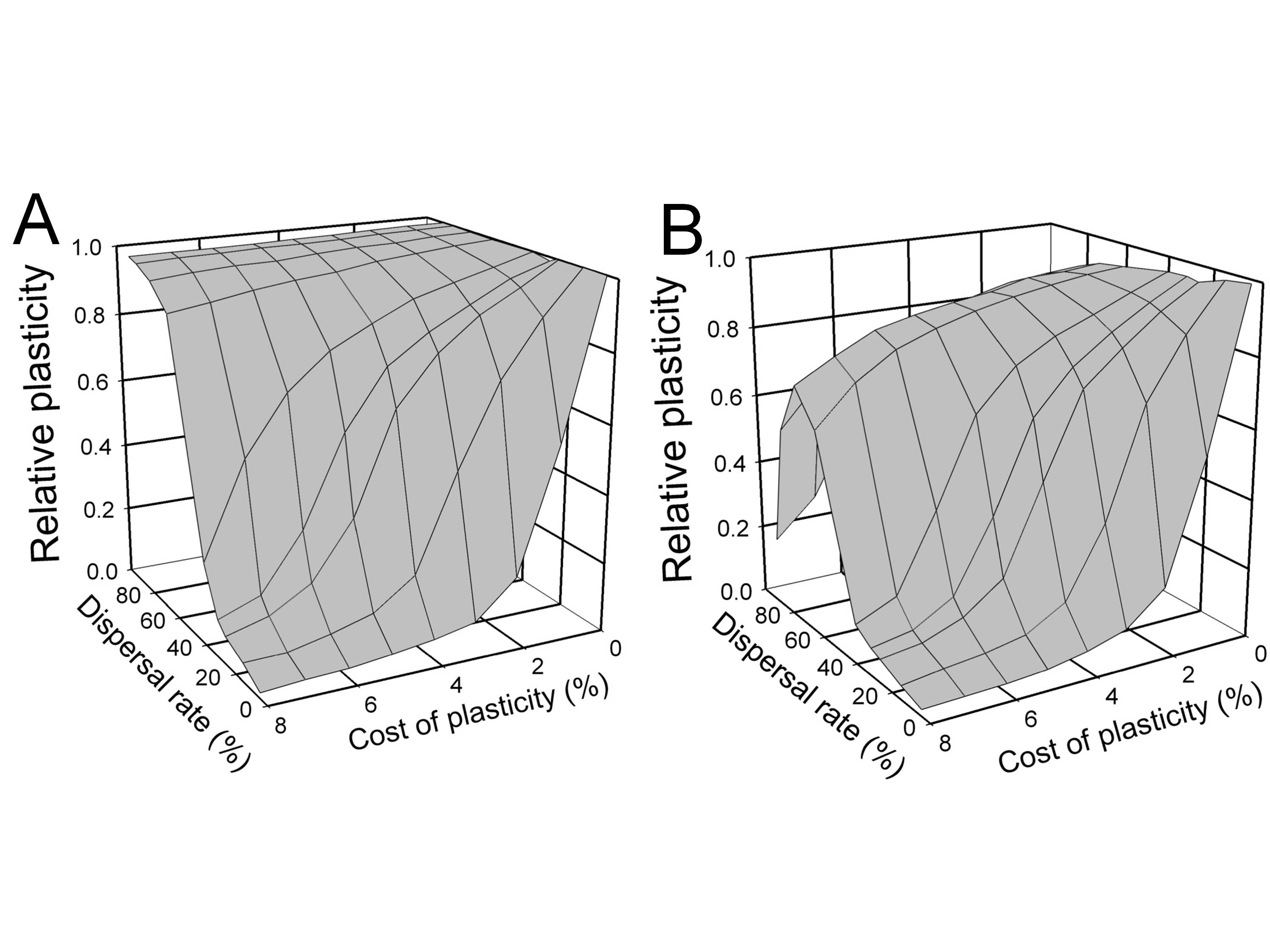


Figure A2. Examples of the transient behavior of plasticity (black) and dispersal rate (red) for different values of initial relative plasticity and dispersal rate without linkage. In these simulations dispersal occurred before selection (move first) and the cost of optimal plasticity was 2%.


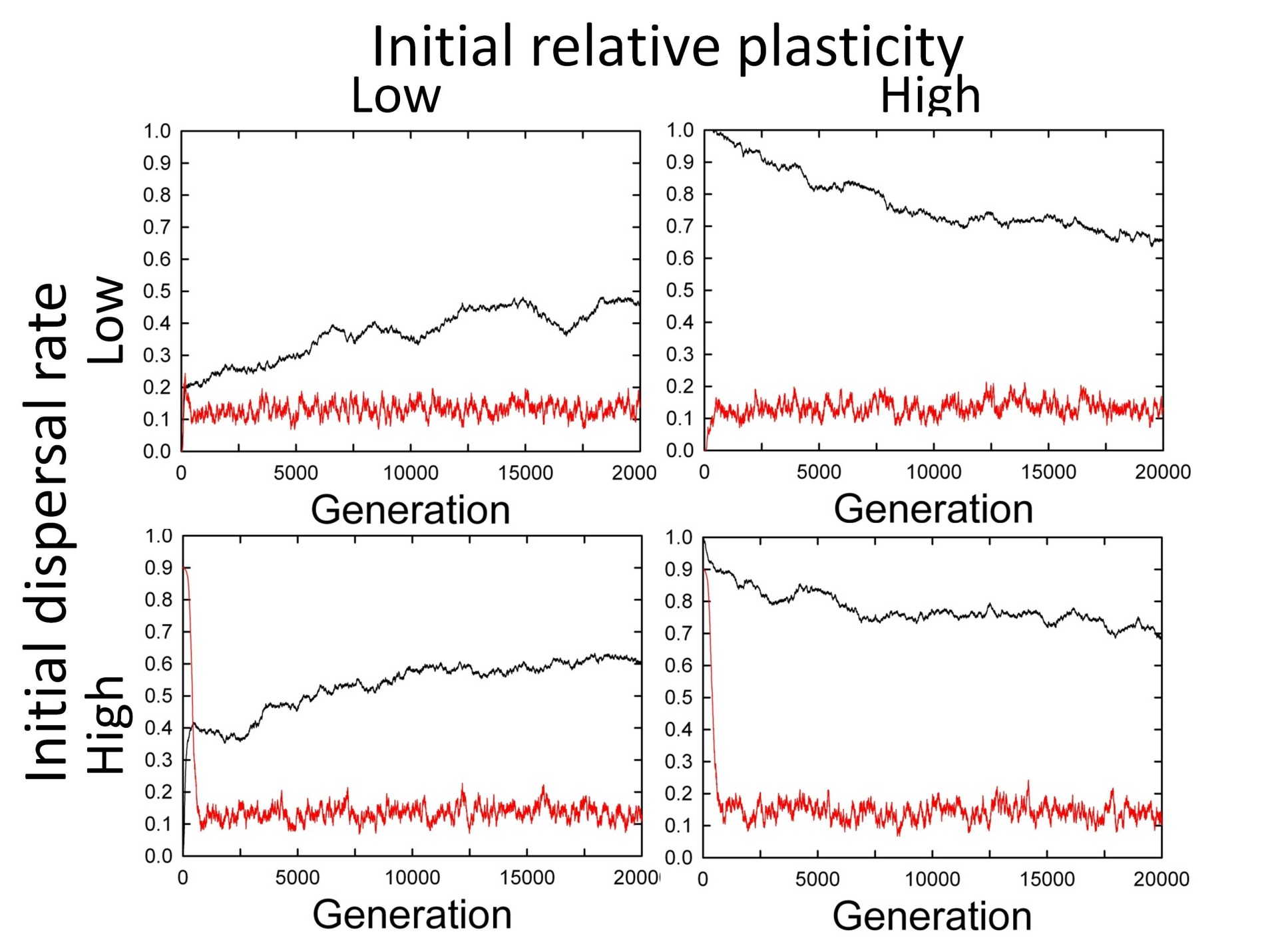

Supplement: Supplementary file 1 [file ece30002-2027-SD1.docx]
