# Supplementary material for: The genetics of phenotypic plasticity. XI. Joint evolution of plasticity and dispersal rate
Source: Ecol Evol. 2012 Jul 20;2(8):2027–39. doi: 10.1002/ece3.327 (PMC3434005; doi:10.1002/ece3.327)
Supplement: Supplementary file 2 [file ece30002-2027-SD2.docx]

Appendix B: Computer code used for the simulations (copyright 2012 Samuel M. Scheiner). This program is free software: you can redistribute it and/or modify it under the terms of the GNU General Public License as published by the Free Software Foundation, either version 3 of the License, or (at your option) any later version. This program is distributed in the hope that it will be useful, but WITHOUT ANY WARRANTY; without even the implied warranty of MERCHANTABILITY or FITNESS FOR A PARTICULAR PURPOSE. See the GNU General Public License for more details <http://www.gnu.org/licenses/>.

! ******** SET ARRAYS AND PARAMETER VECTORS *********

REAL OPT[ALLOCATABLE](:) !Optimal phenotype in each deme

REAL STS[ALLOCATABLE](:) !Standard deviation of selection function in each deme

REAL RALL[ALLOCATABLE](:,:,:) !Initial random allelic values

REAL EOPT[ALLOCATABLE](:) !Optimal phenotype with temporal variation

REAL B[ALLOCATABLE](:) !Environmental effect on plasticity allele in each deme

REAL OUTS[ALLOCATABLE](:,:) !Output variable for each deme

REAL GENO[ALLOCATABLE](:,:,:) !Individuals

REAL POPMEAN[ALLOCATABLE](:,:) !Mean population values

REAL YOUNG[ALLOCATABLE](:,:) !Offspring

REAL MEANFIT !Mean fitness of the metapopulation

REAL MR !Initial migration rate

REAL MUTRATE !Mutation rate of plastic and nonplastic loci

REAL MUTVAR !Variance of mutation function of plastic and nonplastic loci

REAL MMFIT !Sum of mean fitness values across replicates

REAL MGMUT !Mutation rate of dispersal loci

REAL MIGVAR !Variance of mutation function of dispersal loci

REAL MBIAS !Initial allelic bias of nonplastic loci

REAL MIGR(10) !Vector of migration rates

REAL GRS(9) !Vector of slope values of continuous environmental gradient

REAL GRSTEP(4) !Vector of step values of stepped environmental gradient

REAL STE(7) !Vector of slope values of the effects of the environment on the plasticity loci

REAL MBIS(9) !Vector of initial allelic bias of nonplastic loci

REAL LINKAGE !Amount of genetic linkage

REAL LNK(5) !Vector of amount of genetic linkage

REAL PLSTEP(4) !Vector of step values of the effects of the environment on the plasticity loci

REAL ENO(12) !Vector of the amount of environmental noise at the time of selection

REAL COSTER(13) !Vector of cost of plasticity values

REAL CNO(7) !Vector of autocorrelation of environmental noise

DOUBLE PRECISION RSUM[ALLOCATABLE](:,:) !Sum of means of deme parameters across replicates

DOUBLE PRECISION SUMSQ[ALLOCATABLE](:,:) !Sum of squares of means of deme parameters across replicates

INTEGER MCOUNT[ALLOCATABLE](:) !Number of individuals in a deme before selection

INTEGER NCOUNT[ALLOCATABLE](:) !Number of individuals in a deme after selection

INTEGER NQ1[ALLOCATABLE](:) !Queue for tracking individuals during mortality

INTEGER NQ2[ALLOCATABLE](:) !Queue for tracking individuals during mortality

INTEGER GRD !Counter for environmental gradient vector

INTEGER STP !Counter for reaction norm vector

INTEGER SEED !Seed for random number generator

INTEGER BIAS !Initial allelic bias for plastic loci

INTEGER VMLOCI !Initial genetic variation for nonplastic loci

INTEGER VPLLOCI !Initial genetic variation for plastic loci

INTEGER TGEN !Number of generations per replicate

INTEGER*4 MRL !Counter for migration rate vector

INTEGER*4 I !Deme ID

INTEGER*4 J !Individual ID

INTEGER*4 K !Allele ID

CHARACTER GTYPE*4 !Shape of environmental gradient

CHARACTER PLTYPE*4 !Shape of reaction norm

DATA MIGR /0,0.05,0.07,0.09,0.1,0.12,0.2,0.3,0.5,0.8/

DATA ENO /0,0.05,0.1,0.15,0.2,0.25,0.3,0.35,0.4,0.45,0.5,0.55/

DATA CNO /0.75,0.5,0.25,0,-0.25,-0.5,-0.75/

DATA COSTER /0,0.0001,0.0002,0.0003,0.0004,0.0005,0.0006,

C 0.0007,0.0008,0.0009,0.0010,0.0011,0.0012/

DATA GRS /0.0,0.1,0.2,0.3,0.4,0.5,0.6,0.7,0.8/

DATA GRSTEP /0,5,10,15/

DATA STE /0,0.01,0.02,0.03,0.04,0.05,0.06/

DATA PLSTEP /0,5,10,15/

DATA MBIS /0,0.25,0.5,0.75,1,1.25,1.5,1.75,2/

DATA LNK /0,0.25,0.5,0.75,1/

!

!

! ******* SET OUTPUT FILE AND GET SIMULATION PARAMETERS ***************

CALL INOUT

CALL TRANSOUT

CALL SIMPARAM(NDEME,GTYPE,PLTYPE,NPOP,MAXPOP,NREP,MAXCRASH,

C TGEN,OPTMEAN,OFFSET,WIDTH,MUTRATE,MGMUT,

C MUTVAR,MIGVAR,SEED,NPARAM)

!

! ******* WRITE SIMULATION PARAMETER METADATA TO OUTPUT FILES **********

CALL TRANSMETA(NDEME,GTYPE,PLTYPE,NPOP,MAXPOP,NREP,OPTMEAN,

C OFFSET,WIDTH,MUTRATE,MUTVAR,MGMUT,MIGVAR)

CALL METADATA(NDEME,GTYPE,PLTYPE,NPOP,MAXPOP,NREP,TGEN,

C OPTMEAN,OFFSET,WIDTH,MUTRATE,MUTVAR,MGMUT,MIGVAR)

!

! ************ DIMENSION ARRAYS ***************

ALLOCATE (OPT(NDEME),STS(NDEME),B(NDEME),OUTS(NDEME,(2*NPARAM)),

C RSUM(NDEME,NPARAM),MCOUNT(NDEME),NCOUNT(NDEME),

C POPMEAN(NDEME,NPARAM),SUMSQ(NDEME,NPARAM),NQ1(MAXPOP),

C NQ2(MAXPOP),EOPT(NDEME))

!

! ******* BEGIN LOOPING PARAMETER COMBINATIONS ********

!

! ******* SET LIFE HISTORY *******

! ******* SET TYPE OF SELECTIVE REPRODUCTION (1 = soft, 2 = hard)

DO 25 NSH = 1,1

! ******* SET MOVEMENT-SELECTION ORDER (1 = move first, 2 = select first) ******

DO 24 MS = 1,2

! ******* ESTABLISH GENOMIC ARCHITECTURE *******

! ******* NMLOCI = NUMBER OF MEAN LOCI ******

DO 19 NMLOCI = 1,1

! ******* NPLOCI = NUMBER OF PLASTICITY LOCI ******

DO 18 NPLOCI = 1,1

! ******* NMIGL = NUMBER OF MIGRATION LOCI ******

DO 17 NMIGL = 1,1

! ******* TOTAL NUMBER OF LOCI ******

NMALL = 2*NMLOCI

NPALL = 2*NPLOCI

NPHALL = NMALL + NPALL

NMGALL = 2*NMIGL

NALL = NMALL + NPALL + NMGALL

! ******* PHENOTYPE MEASURES ********

NMEAN = NALL + 1

NPLAST = NALL + 2

NMIGR = NALL + 3

NPHENO = NALL + 4

NLIVE = NALL + 5

! ******* DIMENSION ALLELIC AND GENOME ARRAYS ********

ALLOCATE (RALL(NDEME,NALL,5),YOUNG(MAXPOP,NALL),

C GENO(NDEME,MAXPOP,NLIVE))

!

! ******* SET GENOMIC PARAMETERS *******

! ******* VMLOCI = INITIAL GENETIC VARIATION OF MEAN LOCI (0 or 1) *****

DO 23 VMLOCI = 0,0

! ******* MPLLOCI = INITIAL GENETIC VARIAITON OF PLASTIC LOCI (0 or 1) ******

DO 22 VPLLOCI = 0,0

! ******* LINKAGE = GENETIC LINKAGE (1 = none, 5 = complete) *******

DO 21 LIN = 1,5,4

LINKAGE = LNK(LIN)

! ******* MBIAS = ALLELIC BIAS TOWARDS OPTIMAL MEAN (1 = none) ******

DO 20 MBI = 1,1

MBIAS = MBIS(MBI)

! ******* BIAS = ALLELIC BIAS TOWARDS OPTIMAL PLASTICITY (0 = none) ******

DO 16 BIAS = 0,7

!

! ******* SET ENVIRONMENTAL GRADIENT PARAMETERS *******

! ******* GRAD = SLOPE OR STEP OF OPTIMAL PHENOTYPE FUNCTION *****

DO 15 GRD = 5,5

IF (GTYPE.EQ.'CONT') THEN

GRAD = GRS(GRD)

ELSE

GRAD = GRSTEP(GRD)

END IF

! ****** IF ENVIRONMENT = STEP, SET NUMBER OF STEPS ******

DO 151 NSTEP = 1,1

! ****** SLOPE = SLOPE OR STEP OF PLASTICITY FUNCTION ********

DO 13 STP = 5,5

IF (PLTYPE.EQ.'CONT') THEN

SLOPE = STE(STP)

ELSE

SLOPE = PLSTEP(STP)

END IF

! ****** SET SELECTION GRADIENT AND ALLELIC VALUES *******

CALL INITIAL(GRAD,OPT,EOPT,STS,RALL,B,SLOPE,GTYPE,PLTYPE,

C OPTMEAN,OFFSET,WIDTH,NSTEP,NDEME,NALL,NMALL,

C NPHALL,NPALL,MBIAS,BIAS,VMLOCI,VPLLOCI)

!

! ****** SET UNPREDICTABILITY PARAMETERS *******

! ****** RNOISE = VARIANCE OF ENVIRONMENTAL NOISE AT SELECTION (1 = none) *******

DO 111 ENS = 1,1

ENOISE = ENO(ENS) !Set variance of environmental noise

RNOISE = ENOISE*GRAD*NDEME !Relativize to gradient

! ****** CNOISE = AUTOCORRELATION OF ENVIRONMENTAL NOISE AT SELECTION (4 = none) *******

DO 112 CNS = 4,4

CNOISE = CNO(CNS) !Set autocorrelation of environmental noise

! ****** COST = COST OF PLASTICITY (1 = none) *******

DO 113 CST = 1,9

COST = COSTER(CST) !Set cost of plasticity

RCOST = COST*GRAD/SLOPE !Relativize to gradient

! ****** MR = INITIAL MIGRATION RATE ********

DO 11 MRL = 1,10

MR = MIGR(MRL)*(5.0/NMIGL)

!

! **************** BEGIN BODY OF PROGRAM *********************

MMFIT = 0

DO 3 I = 1,NDEME

DO 3 J = 1,NPARAM

RSUM(I,J) = 0

3 SUMSQ(I,J) = 0

! ********** BEGIN REPLICATES FOR ONE PARAMETER COMBINATION ********

NCRASH = 0

MREP = 0

81 MREP = MREP + 1

CALL POPS(NPOP,RALL,B,GENO,MCOUNT,NDEME, !Initialize metapopulation

C MR,MAXPOP,NMEAN,NPLAST,NMIGR,

C NPHENO,NLIVE,NALL,NMALL,NPALL,

C NPHALL,SEED)

DO 2 NGEN = 1,TGEN

IF (NSH.EQ.1) CALL REPSOFT(NPOP,MCOUNT,GENO, !Soft selection reproduction

C YOUNG,NDEME,MAXPOP,NALL,NLIVE,LINKAGE)

IF (NSH.EQ.2) THEN !Hard selection

CALL MORTAL(MCOUNT,GENO,NDEME,MAXPOP,NLIVE, !Reduce deme to carrying capacity

C NPOP,NQ1,NQ2)

CALL REPHARD(MCOUNT,GENO,YOUNG,NDEME,MAXPOP, !Fixed fecundity reproduction

C NALL,NLIVE,LINKAGE)

END IF

CALL MUTATE(MCOUNT,GENO,NDEME,MAXPOP,NALL, !Mutate alleles

C NPHALL,NLIVE,MUTRATE,MGMUT,MUTVAR,MIGVAR)

CALL DEVELOP(MCOUNT,B,GENO,NDEME,MAXPOP,NALL, !Determine phenotype

C NMALL,NPHALL,NMEAN,NPLAST,NMIGR,

C NPHENO,NLIVE)

CALL FITS(MCOUNT,GENO,STS,OPT,MEANFIT, !Calculate metapop mean fitness

C NDEME,MAXPOP,NPLAST,NPHENO,NLIVE,RCOST)

IF (MEANFIT.EQ.0.0) THEN

MREP = MREP - 1

NCRASH = NCRASH + 1

IF (MREP.EQ.0) THEN

IF (NCRASH.EQ.MAXCRASH) GOTO 82

END IF

GOTO 81

END IF

IF (MS.EQ.1) CALL MIGRATE(MCOUNT,NCOUNT,GENO, !Move before selection

C NDEME,MAXPOP,NALL,NMIGR,NLIVE)

CALL SELECT(MCOUNT,GENO,STS,OPT,NDEME,MAXPOP, !Selection

C NALL,NPLAST,NPHENO,NLIVE,RNOISE,

C RCOST,EOPT,CNOISE)

IF (MS.EQ.2) CALL MIGRATE(MCOUNT,NCOUNT,GENO, !Move after selection

C NDEME,MAXPOP,NALL,NMIGR,NLIVE)

CALL WHEREAT(NSH,MS,GRAD,SLOPE,MBIAS,BIAS,MR,

C NGEN,MEANFIT,MREP,NMLOCI,NPLOCI,NMIGL,

C LINKAGE,VMLOCI,VPLLOCI,CNOISE,ENOISE,COST)

IF (MREP.EQ.1) THEN !Output transient of 1st replicate

CALL TRANSIENT(NSH,MS,GRAD,SLOPE,BIASM,BIAS,MR,

C NGEN,NMLOCI,NPLOCI,NMIGL,LINKAGE,VMLOCI,

C VPLLOCI,MCOUNT,GENO,NDEME,MAXPOP,NMEAN,

C NPLAST,NMIGR,NPHENO,NLIVE,STS,OPT,

C MEANFIT,ENOISE,COST,CNOISE)

END IF

2 CONTINUE

! ******** MEASURE PARAMETERS FOR THAT REPLICATE ********

4 CALL MEASURE(MCOUNT,GENO,POPMEAN,NDEME,

C MAXPOP,NALL,NMALL,NPALL,NPHALL,NMGALL,

C NMEAN,NPLAST,NMIGR,NPHENO,NLIVE,STS,

C OPT,NPARAM)

! ********* CALCULATE SUMS AND SUMS-OF-SQUARES OF PARAMETERS ********

DO 6 I = 1,NDEME

DO 5 J = 1,NPARAM

RSUM(I,J) = RSUM(I,J) + POPMEAN(I,J)

SUMSQ(I,J)=SUMSQ(I,J)+(POPMEAN(I,J)*POPMEAN(I,J))

5 CONTINUE

6 CONTINUE

MMFIT = MMFIT + MEANFIT

8 IF (MREP.LT.NREP) GOTO 81

! ****** CALCULATE MEAN AND SE VALUES FOR THIS PARAMETER COMBINATION ******

82 DO 9 I = 1,NDEME

DO 9 J = 1,NPARAM

JJ = (J*2)-1

OUTS(I,JJ) = RSUM(I,J)/(NREP*1.0)

OUTS(I,(J*2)) = SE(RSUM(I,J),SUMSQ(I,J),NREP)

9 CONTINUE

AMFIT = MMFIT/(NREP*1.0)

! ********* OUTPUT RESULTS FOR THIS PARAMETER COMBINATION *********

CALL OUTER(NSH,MS,GRAD,SLOPE,MR,OUTS,NDEME,AMFIT,

C NCRASH,NPARAM,MBIAS,BIAS,NMLOCI,NPLOCI,NMIGL,

C LINKAGE,VMLOCI,VPLLOCI,ENOISE,COST,CNOISE)

! *********** LOOP TO NEW PARAMETER COMBINATION **********

11 CONTINUE

113 CONTINUE

112 CONTINUE

111 CONTINUE

13 CONTINUE

151 CONTINUE

15 CONTINUE

16 CONTINUE

20 CONTINUE

21 CONTINUE

22 CONTINUE

23 CONTINUE

DEALLOCATE (RALL,YOUNG,GENO)

17 CONTINUE

18 CONTINUE

19 CONTINUE

24 CONTINUE

25 CONTINUE

STOP

END

!

!

!

! ******* THIS SUBROUTINE SETS THE PARAMETER VALUES FOR THE SIMULATION *******

SUBROUTINE SIMPARAM(NDEME,GTYPE,PLTYPE,NPOP,MAXPOP,NREP,

C MAXCRASH,TGEN,OPTMEAN,OFFSET,WIDTH,

C MUTRATE,MGMUT,MUTVAR,MIGVAR,SEED,NPARAM)

REAL MUTRATE,MUTVAR,MGMUT,MIGVAR

INTEGER SEED,TGEN

CHARACTER GTYPE*4,PLTYPE*4

! ****** CHANGE "SEED" TO OBTAIN DIFFERENT RANDOM NUMBER *******

! ****** SEQUENCE. IT MUST BE A LARGE, ODD NUMBER. *******

SEED = 12231957

!

! ************ SET PARAMETERS FOR DESIRED MODELS ***********

!

! ****** GTYPE = TYPE OF GRADIENT (CONT(INUOUS) OR STEP) ********

GTYPE = 'CONT'

! ****** PLTYPE = TYPE OF REACTION NORM (CONT(INUOUS) OR STEP) ********

PLTYPE = 'CONT'

! ****** SET NUMBER OF DEMES ******

NDEME = 50

! ****** NPOP = POPULATION SIZE/CARRYING CAPACITY ****

NPOP = 100

! ****** MAXPOP = MAXIMUM POPULATION SIZE *****

MAXPOP = 4*NPOP

! ******** NREP = NUMBER OF REPLICATES PER PARAMTER SET ******

NREP = 20

! ******** MAXCRASH = MAXIMUM NUMBER OF POPULATIONS CRASHES ******

MAXCRASH = 3*NREP

! ******** TGEN = TOTAL NUMBER OF GENERATIONS PER REPLICATE ******

TGEN = 20000

! ****** OPTMEAN = OFFSET OF GRADIENT ALONG Y AXIS *******

OPTMEAN = 0

! ******* OFFSET = OFFSET OF GRADIENT ALONG X AXIS *******

OFFSET = 0

! ******* WIDTH = WIDTH OF THE SELECTION FUNCTION *******

WIDTH = 2

! ******* MUTRATE = PHENOTYPE MUTATION RATE *******

MUTRATE = 0.1

! ******* MGMUT = MIGRATION MUTATION RATE *******

MGMUT = 0.1

! ******* MUTVAR = STANDARD DEVIATION OF PHENOTYPE MUTATION FUNCTION *****

MUTVAR = 0.1

! ******* MIGVAR = STANDARD DEVIATION OF MIGRATION MUTATION FUNCTION *****

MIGVAR = 0.1

! ******* NPARAM = NUMBER OF PARAMETERS PRODUCED BY SIMULATION *****

NPARAM = 16

RETURN

END

!

!

! ****THIS SUBROUTINE INPUTS SELECTION GRADIENT AND ALLELIC VALUES ****

SUBROUTINE INITIAL(GRAD,OPT,EOPT,STS,RALL,B,SLOPE,GTYPE,PLTYPE,

C OPTMEAN,OFFSET,WIDTH,NSTEP,NDEME,NALL,NMALL,

C NPHALL,NPALL,MBIAS,PLBIAS,VMLOCI,VPLLOCI)

REAL OPT(NDEME),STS(NDEME),RALL(NDEME,NALL,5),SETALL(5),B(NDEME)

REAL MBIAS,EOPT(NDEME)

INTEGER*4 I,J

INTEGER PLBIAS,VMLOCI,VPLLOCI

CHARACTER*4 GTYPE,PLTYPE

DATA SETALL /-2,-1,0,1,2/

CENT = (-1.0*((NDEME+1.0)/2.0))

! ******** SET SELECTION FUNCTION *******

IF (GTYPE.EQ.'CONT') THEN

XI = 1.0 !Continuous gradients

DO 8 I = 1,NDEME

OPT(I)= OPTMEAN + (GRAD * (CENT + OFFSET + XI))

EOPT(I) = OPT(I)

8 XI = XI + 1.0

ELSE

ND = NDEME/(2*NSTEP) !Step gradients

DO 13 K = 1,(NSTEP+1)

NDF = (K-1)*ND + 1 !Determine first deme for a given step

NDL = K*ND !Determine last deme for a given step

OPTIMUM = OPTMEAN+(GRAD*(K - 1.0))-((GRAD*NSTEP)/2.0)

DO 13 I = NDF,NDL

EOPT(I) = OPTIMUM

13 OPT(I) = OPTIMUM

END IF

! ****** SET WIDTH OF SELECTION FUNCTION IN EACH ENVIRONMENT ******

DO 9 I = 1,NDEME

9 STS(I) = WIDTH

! ****** SET INITIAL ALLELIC VALUES ******

DO 14 I = 1,NDEME

DO 10 K = 1,NMALL

DO 10 J=1,5

10 RALL(I,K,J) = VMLOCI*(SETALL(J)) + (MBIAS*OPT(I))

DO 12 K = (NMALL+1),NPHALL

DO 12 J=1,5

12 RALL(I,K,J) = VPLLOCI*(SETALL(J)) + PLBIAS

14 CONTINUE

! ****** SET REACTION NORM FUNCTION *******

IF (PLTYPE.EQ.'CONT') THEN

XI = 1.0 !Continuous function

DO 11 I = 1,NDEME

B(I) = SLOPE * (CENT + OFFSET + XI)

11 XI = XI + 1.0

ELSE

ND = NDEME/(2*NSTEP) !Step function

DO 15 K = 1,(NSTEP+1)

NDF = (K-1)*ND + 1 !Determine first deme for a given step

NDL = K*ND !Determine last deme for a given step

PLASTEP = K - 1.0 - (NSTEP/2.0)

DO 15 I = NDF,NDL

15 B(I) = PLASTEP

END IF

RETURN

END

!

!

!

! ****THIS SUBROUTINE INITIALIZES THE POPULATION COMPOSITION ****

SUBROUTINE POPS(N,RALL,B,GENO,MCOUNT,NDEME,MR,MAXPOP,

C NMEAN,NPLAST,NMIGR,NPHENO,NLIVE,

C NALL,NMALL,NPALL,NPHALL,SEED)

REAL RALL(NDEME,NALL,5),B(NDEME),GENO(NDEME,MAXPOP,NLIVE),MEAN,MR

INTEGER MCOUNT(NDEME),SEED

INTEGER*4 I,J,K

! ******* FOR THE Jth INDIVIDUAL IN THE Ith DEME ********

DO 25 I = 1,NDEME

DO 24 J = 1,N

! ******* CREATE A RANDOM GENOTYPE *********

DO 21 K = 1,NPHALL

L = INT((5.0*RAN(SEED)) + 1.0)

GENO(I,J,K) = RALL(I,K,L)

21 CONTINUE

DO 27 K=(NPHALL+1),NALL

GENO(I,J,K) = MR

27 CONTINUE

! ******* DETERMINE THE PHENOTYPE ********

MEAN = 0

DO 22 K = 1,NMALL

22 MEAN = MEAN + GENO(I,J,K) !Sum the non-plastic loci

PLAST = 0

DO 23 K = (NMALL+1),NPHALL

23 PLAST = PLAST + GENO(I,J,K) !Sum the plastic loci

PMIG = 0

DO 26 K = (NPHALL+1),NALL

26 PMIG = PMIG + GENO(I,J,K) !Sum the migration loci

GENO(I,J,NMEAN) = MEAN

GENO(I,J,NPLAST) = PLAST

GENO(I,J,NMIGR) = PMIG

GENO(I,J,NPHENO) = MEAN + (B(I)*PLAST)

GENO(I,J,NLIVE) = 1.0

24 CONTINUE

MCOUNT(I) = N !Set initial population size

25 CONTINUE

RETURN

END

!

!

!

! *******THIS SUBROUTINE PERFORMS GAUSSIAN SELECTION *****

SUBROUTINE SELECT(MCOUNT,GENO,STS,OPT,NDEME,MAXPOP,

C NALL,NPLAST,NPHENO,NLIVE,RNOISE,RCOST,EOPT,CNOISE)

REAL GENO(NDEME,MAXPOP,NLIVE),STS(NDEME),OPT(NDEME),EOPT(NDEME)

INTEGER MCOUNT(NDEME),SEED

INTEGER*4 I,J

H = -0.5

DO 32 I = 1,NDEME

EOLD = EOPT(I)

EOPT(I) = OPT(I)+(CNOISE*(EOLD-OPT(I)))+ !Randomly vary deme environment

C (RNOISE*ZMUT(SEED)*SQRT(1-(CNOISE*CNOISE))) !Autocorrelated noise

DO 31 J = 1,MCOUNT(I)

CT = ABS(RCOST*GENO(I,J,NPLAST)) !Cost of plasticity

Z = (GENO(I,J,NPHENO) - EOPT(I))/STS(I)

FITNESS = EXP(H * Z * Z) - CT !Measure fitness

IF (FITNESS.LT.RAN(SEED)) GENO(I,J,NLIVE) = 0 !Decide fate

31 CONTINUE

32 CONTINUE

RETURN

END

!

!

!

! ***** THIS SUBROUTINE PERFORMS SOFT SELECTION REPRODUCTION ******

SUBROUTINE REPSOFT(N,MCOUNT,GENO,YOUNG,NDEME,MAXPOP,

C NALL,NLIVE,LINKAGE)

REAL GENO(NDEME,MAXPOP,NLIVE),YOUNG(MAXPOP,NALL)

REAL LINKAGE

INTEGER MCOUNT(NDEME),SEED

INTEGER*4 I,J,K,K3,K4

IF (LINKAGE.EQ.1.0) THEN

GOTO 1

ELSE IF (LINKAGE.EQ.0.0) THEN

GOTO 2

ELSE

GOTO 3

END IF

!

! ***** Procedure for complete linkage *******

1 CONTINUE

! ******** DETERMINE THAT THERE IS AT LEAST 1 LIVE INDIVIDUAL IN THE POPULATION *******

DO 148 I = 1,NDEME

DO 140 J = 1,MCOUNT(I)

IF (GENO(I,J,NLIVE).EQ.1) GOTO 139

140 CONTINUE

MCOUNT(I) = 0 !If there are no live individuals

GOTO 148

139 XI = MCOUNT(I) !Note population size of Ith deme

! ***** Create offspring by randomly pairing gametes from living individuals ********

DO 144 J = 1,N

! ******* Choose first parent ********

141 JJ = INT((XI*RAN(SEED)) + 1.0) !Pick a random individual

IF (GENO(I,JJ,NLIVE).EQ.0) GOTO 141 !Determine if alive or try again

! ***** Choose one set of linked alleles for first gamete ******

IF (RAN(SEED).GT.0.5) THEN

LINK = 1

ELSE

LINK = 0

END IF

DO 142 K3 = 1,NALL,2

K4 = K3 + LINK

142 YOUNG(J,K3) = GENO(I,JJ,K4) !Create first half of new genome

! ******* Choose second parent ********

143 JJ = INT((XI*RAN(SEED)) + 1.0) !Pick another random individual

IF (GENO(I,JJ,NLIVE).EQ.0) GOTO 143 !Determine if alive or try again

! ******* Choose one of linked alleles for other gamete ********

IF (RAN(SEED).GT.0.5) THEN

LINK = 0

ELSE

LINK = -1

END IF

DO 149 K3 = 2,NALL,2

K4 = K3 + LINK

149 YOUNG(J,K3) = GENO(I,JJ,K4) !Create second half of new genome

144 CONTINUE

! ******* Create next generation from gametes *******

! ******* This step restores the population size to the carrying capacity ******

DO 147 J = 1,N

DO 145 K = 1,NALL

145 GENO(I,J,K) = YOUNG(J,K)

147 CONTINUE

MCOUNT(I) = N !Record population size

148 CONTINUE

RETURN !End complete linkage and return

!

! ****** Procedure for no linkage *******

2 CONTINUE

! ******** DETERMINE THAT THERE IS AT LEAST 1 LIVE INDIVIDUAL IN THE POPULATION *******

DO 48 I = 1,NDEME

DO 40 J = 1,MCOUNT(I)

IF (GENO(I,J,NLIVE).EQ.1) GOTO 39

40 CONTINUE

MCOUNT(I) = 0 !If there are no live individuals

GOTO 48

39 XI = MCOUNT(I) !Note population size of Ith deme

! ***** Create offspring by randomly pairing gametes from living individuals ********

DO 44 J = 1,N

! ******* Choose first parent ********

41 JJ = INT((XI*RAN(SEED)) + 1.0) !Pick a random individual

IF (GENO(I,JJ,NLIVE).EQ.0) GOTO 41 !Determine if alive or try again

! ******* Choose one of each allele pair for first gamete ********

DO 42 K3 = 1,NALL,2

IF (RAN(SEED).GT.0.5) THEN

K4 = K3 + 1

ELSE

K4 = K3

END IF

42 YOUNG(J,K3) = GENO(I,JJ,K4) !Create first half of new genome

! ******* Choose second parent ********

43 JJ = INT((XI*RAN(SEED)) + 1.0) !Pick another random individual

IF (GENO(I,JJ,NLIVE).EQ.0) GOTO 43 !Determine if alive or try again

! ******* Choose one of each allele pair for other gamete ********

DO 49 K3 = 2,NALL,2

IF (RAN(SEED).GT.0.5) THEN

K4 = K3 - 1

ELSE

K4 = K3

END IF

49 YOUNG(J,K3) = GENO(I,JJ,K4) !Create second half of new genome

44 CONTINUE

! ******* Create next generation from gametes *******

! ******* This step restores the population size to the carrying capacity ******

DO 47 J = 1,N

DO 45 K = 1,NALL

45 GENO(I,J,K) = YOUNG(J,K)

47 CONTINUE

MCOUNT(I) = N !Record population size

48 CONTINUE

RETURN !End no linkage and return

!

! ***** Procedure for partial linkage *******

3 CONTINUE

LINK = 0.5 + (LINKAGE/2.0)

! ******** DETERMINE THAT THERE IS AT LEAST 1 LIVE INDIVIDUAL IN THE POPULATION *******

DO 348 I = 1,NDEME

DO 340 J = 1,MCOUNT(I)

IF (GENO(I,J,NLIVE).EQ.1) GOTO 339

340 CONTINUE

MCOUNT(I) = 0 !If there are no live individuals

GOTO 348

339 XI = MCOUNT(I) !Note population size of Ith deme

! ***** Create offspring by randomly pairing gametes from living individuals ********

DO 344 J = 1,N

! ******* Choose first parent ********

341 JJ = INT((XI*RAN(SEED)) + 1.0) !Pick a random individual

IF (GENO(I,JJ,NLIVE).EQ.0) GOTO 341 !Determine if alive or try again

! ***** Choose one set of linked alleles for first gamete ******

K5 = 1

DO 342 K3 = 1,NALL,2

IF (K5.EQ.1) THEN

IF (RAN(SEED).GT.LINK) THEN

K4 = K3 + 1

K5 = MOD(K4,2)

ELSE

K4 = K3

K5 = MOD(K4,2)

END IF

ELSE

IF (RAN(SEED).GT.LINK) THEN

K4 = K3

K5 = MOD(K4,2)

ELSE

K4 = K3 + 1

K5 = MOD(K4,2)

END IF

END IF

342 YOUNG(J,K3) = GENO(I,JJ,K4) !Create first half of new genome

! ******* Choose second parent ********

343 JJ = INT((XI*RAN(SEED)) + 1.0) !Pick another random individual

IF (GENO(I,JJ,NLIVE).EQ.0) GOTO 343 !Determine if alive or try again

! ******* Choose one of linked alleles for other gamete ********

K5 = 0

DO 349 K3 = 2,NALL,2

IF (K5.EQ.1) THEN

IF (RAN(SEED).GT.LINK) THEN

K4 = K3

K5 = MOD(K4,2)

ELSE

K4 = K3 - 1

K5 = MOD(K4,2)

END IF

ELSE

IF (RAN(SEED).GT.LINK) THEN

K4 = K3 - 1

K5 = MOD(K4,2)

ELSE

K4 = K3

K5 = MOD(K4,2)

END IF

END IF

349 YOUNG(J,K3) = GENO(I,JJ,K4) !Create second half of new genome

344 CONTINUE

! ******* Create next generation from gametes *******

! ******* This step restores the population size to the carrying capacity ******

DO 347 J = 1,N

DO 345 K = 1,NALL

345 GENO(I,J,K) = YOUNG(J,K)

347 CONTINUE

MCOUNT(I) = N !Record population size

348 CONTINUE

RETURN !End partial linkage and return

END

!

!

!

!****** THIS SUBROUTINE REDUCES THE DEME SIZE TO THE CARRYING CAPACITY *******

SUBROUTINE MORTAL(MCOUNT,GENO,NDEME,MAXPOP,NLIVE,NPOP,NQ1,NQ2)

REAL GENO(NDEME,MAXPOP,NLIVE)

INTEGER MCOUNT(NDEME),NQ1(MAXPOP),NQ2(MAXPOP),SEED

INTEGER*4 I,J,K,N

DO 1 I = 1,NDEME

HLIVE = 0

DO 6 J = 1,MCOUNT(I)

6 IF (GENO(I,J,NLIVE).EQ.1) HLIVE = HLIVE + 1 !Count live individuals

IF (HLIVE.LE.NPOP) GOTO 1

IND = 0

DO 2 J = 1,MCOUNT(I)

IF (GENO(I,J,NLIVE).EQ.0) GOTO 2 !Skip dead individuals

IND = IND + 1

NQ1(IND) = J !Create picking queue

GENO(I,J,NLIVE) = 0 !Set all live individuals to "dead"

2 CONTINUE

DO 3 K = 1,NPOP !Set population to carrying capacity

NP = INT(IND*RAN(SEED) + 1.0)

GENO(I,NQ1(NP),NLIVE) = 1.0 !Resurrect chosen individual

N = 0

DO 4 KK = 1,IND

IF (KK.EQ.NP) GOTO 4

N = N + 1

NQ2(N) = NQ1(KK) !Create new queue of remaining individuals

4 CONTINUE

IND = IND - 1 !Decrement number of remaining individuals

DO 5 KK = 1,IND

5 NQ1(KK) = NQ2(KK) !Replace old queue with new queue

3 CONTINUE

1 CONTINUE

RETURN

END

!

!

!

! ***** THIS SUBROUTINE PERFORMS HARD SELECTION REPRODUCTION ******

SUBROUTINE REPHARD(MCOUNT,GENO,YOUNG,NDEME,MAXPOP,

C NALL,NLIVE,LINKAGE)

REAL GENO(NDEME,MAXPOP,NLIVE),YOUNG(MAXPOP,NALL)

REAL LINKAGE

INTEGER MCOUNT(NDEME),SEED

INTEGER*4 I,J,JN,JJ,JJJ,K,K3,K4

IF (LINKAGE.EQ.1.0) THEN

GOTO 1

ELSE IF (LINKAGE.EQ.0.0) THEN

GOTO 2

ELSE

GOTO 3

END IF

!

! ***** Procedure for complete linkage *******

1 CONTINUE

! ******** DETERMINE THAT THERE IS AT LEAST 1 LIVE INDIVIDUAL IN THE POPULATION *******

DO 148 I = 1,NDEME

DO 140 J = 1,MCOUNT(I)

IF (GENO(I,J,NLIVE).EQ.1) GOTO 139

140 CONTINUE

MCOUNT(I) = 0 !If there are no live individuals

GOTO 148

139 XI = MCOUNT(I) !Note population size of Ith deme

! ***** Create offspring by pairing gametes from living individuals ********

JJJ = 0

DO 144 J = 1,MCOUNT(I)

IF (GENO(I,J,NLIVE).EQ.0) GOTO 144 !Skip if dead

DO 146 JN = 1,4 !Live individuals have 4 offspring

! ***** Choose one set of linked alleles for first gamete ******

JJJ = JJJ + 1 !Count number of offspring

IF (RAN(SEED).GT.0.5) THEN

LINK = 1

ELSE

LINK = 0

END IF

DO 142 K3 = 1,NALL,2

K4 = K3 + LINK

142 YOUNG(JJJ,K3) = GENO(I,J,K4) !Create first half of new genome

! ******* Choose second parent ********

143 JJ = INT((XI*RAN(SEED)) + 1.0) !Pick a random mate

IF (GENO(I,JJ,NLIVE).EQ.0) GOTO 143 !Determine if alive or try again

! ******* Choose one of linked alleles for other gamete ********

IF (RAN(SEED).GT.0.5) THEN

LINK = 0

ELSE

LINK = -1

END IF

DO 149 K3 = 2,NALL,2

K4 = K3 + LINK

149 YOUNG(JJJ,K3) = GENO(I,JJ,K4) !Create second half of new genome

146 CONTINUE

144 CONTINUE

! ******* Create next generation from gametes *******

DO 147 J = 1,JJJ

DO 145 K = 1,NALL

145 GENO(I,J,K) = YOUNG(J,K)

147 CONTINUE

MCOUNT(I) = JJJ !Record population size

148 CONTINUE

RETURN !End complete linkage and return

!

! ****** Procedure for no linkage *******

2 CONTINUE

! ******** DETERMINE THAT THERE IS AT LEAST 1 LIVE INDIVIDUAL IN THE POPULATION *******

DO 48 I = 1,NDEME

DO 40 J = 1,MCOUNT(I)

IF (GENO(I,J,NLIVE).EQ.1) GOTO 39

40 CONTINUE

MCOUNT(I) = 0 !If there are no live individuals

GOTO 48

39 XI = MCOUNT(I) !Note population size of Ith deme

! ***** Create offspring by pairing gametes from living individuals ********

JJJ = 0

DO 44 J = 1,MCOUNT(I)

IF (GENO(I,J,NLIVE).EQ.0) GOTO 44 !Skip if dead

DO 46 JN = 1,4 !Live individuals have 4 offspring

! ******* Choose one of each allele pair for first gamete ********

JJJ = JJJ + 1 !Count number of offspring

DO 42 K3 = 1,NALL,2

IF (RAN(SEED).GT.0.5) THEN

K4 = K3 + 1

ELSE

K4 = K3

END IF

42 YOUNG(JJJ,K3) = GENO(I,J,K4) !Create first half of new genome

! ******* Choose second parent ********

43 JJ = INT((XI*RAN(SEED)) + 1.0) !Pick a random mate

IF (GENO(I,JJ,NLIVE).EQ.0) GOTO 43 !Determine if alive or try again

! ******* Choose one of each allele pair for other gamete ********

DO 49 K3 = 2,NALL,2

IF (RAN(SEED).GT.0.5) THEN

K4 = K3 - 1

ELSE

K4 = K3

END IF

49 YOUNG(JJJ,K3) = GENO(I,JJ,K4) !Create second half of new genome

46 CONTINUE

44 CONTINUE

! ******* Create next generation from gametes *******

DO 47 J = 1,JJJ

DO 45 K = 1,NALL

45 GENO(I,J,K) = YOUNG(J,K)

47 CONTINUE

MCOUNT(I) = JJJ !Record population size

48 CONTINUE

RETURN !End no linkage and return

!

! ***** Procedure for partial linkage *******

3 CONTINUE

LINK = 0.5 + (LINKAGE/2.0)

! ******** DETERMINE THAT THERE IS AT LEAST 1 LIVE INDIVIDUAL IN THE POPULATION *******

DO 348 I = 1,NDEME

DO 340 J = 1,MCOUNT(I)

IF (GENO(I,J,NLIVE).EQ.1) GOTO 339

340 CONTINUE

MCOUNT(I) = 0 !If there are no live individuals

GOTO 348

339 XI = MCOUNT(I) !Note population size of Ith deme

! ***** Create offspring by randomly pairing gametes from living individuals ********

JJJ = 0

DO 344 J = 1,MCOUNT(I)

IF (GENO(I,J,NLIVE).EQ.0) GOTO 344 !Skip if dead

DO 346 JN = 1,4 !Live individuals have 4 offspring

! ***** Choose one set of linked alleles for first gamete ******

JJJ = JJJ + 1 !Count number of offspring

K5 = 1

DO 342 K3 = 1,NALL,2

IF (K5.EQ.1) THEN

IF (RAN(SEED).GT.LINK) THEN

K4 = K3 + 1

K5 = MOD(K4,2)

ELSE

K4 = K3

K5 = MOD(K4,2)

END IF

ELSE

IF (RAN(SEED).GT.LINK) THEN

K4 = K3

K5 = MOD(K4,2)

ELSE

K4 = K3 + 1

K5 = MOD(K4,2)

END IF

END IF

342 YOUNG(JJJ,K3) = GENO(I,J,K4) !Create first half of new genome

! ******* Choose second parent ********

343 JJ = INT((XI*RAN(SEED)) + 1.0) !Pick another random individual

IF (GENO(I,JJ,NLIVE).EQ.0) GOTO 343 !Determine if alive or try again

! ******* Choose one of linked alleles for other gamete ********

K5 = 0

DO 349 K3 = 2,NALL,2

IF (K5.EQ.1) THEN

IF (RAN(SEED).GT.LINK) THEN

K4 = K3

K5 = MOD(K4,2)

ELSE

K4 = K3 - 1

K5 = MOD(K4,2)

END IF

ELSE

IF (RAN(SEED).GT.LINK) THEN

K4 = K3 - 1

K5 = MOD(K4,2)

ELSE

K4 = K3

K5 = MOD(K4,2)

END IF

END IF

349 YOUNG(JJJ,K3) = GENO(I,JJ,K4) !Create second half of new genome

346 CONTINUE

344 CONTINUE

! ******* Create next generation from gametes *******

DO 347 J = 1,JJJ

DO 345 K = 1,NALL

345 GENO(I,J,K) = YOUNG(J,K)

347 CONTINUE

MCOUNT(I) = JJJ !Record population size

348 CONTINUE

RETURN !End partial linkage and return

END

!

!

!

! ****** THIS SUBROUTINE CALCULATES MEAN FITNESS *****

SUBROUTINE FITS(MCOUNT,GENO,STS,OPT,MEANFIT,

C NDEME,MAXPOP,NPLAST,NPHENO,NLIVE,RCOST)

REAL GENO(NDEME,MAXPOP,NLIVE),STS(NDEME),OPT(NDEME)

REAL MEANFIT

INTEGER MCOUNT(NDEME)

INTEGER*4 I,J

H = -0.5

F2 = 0

DO 52 I = 1,NDEME

XI = MCOUNT(I)

F1 = 0

! ******** Compute fitness of each individual *********

DO 51 J = 1,MCOUNT(I)

CT = ABS(RCOST*GENO(I,J,NPLAST))

Z = (GENO(I,J,NPHENO) - OPT(I))/STS(I)

FITNESS = EXP(H * Z * Z) - CT !Fitness of Jth individual

F1 = F1 + FITNESS !Sum across individuals

51 CONTINUE

IF (XI.EQ.0) THEN

F11 = 0

ELSE

F11 = F1/XI !Compute mean fitness of Ith deme

END IF

F2 = F2 + F11 !Sum across demes

52 CONTINUE

MEANFIT = F2/(1.0*NDEME) !Compute global mean fitness

RETURN

END

!

!

!

! *********THIS SUBROUTINE PERFORMS MUTATION *********

SUBROUTINE MUTATE(MCOUNT,GENO,NDEME,MAXPOP,NALL,NPHALL,

C NLIVE,MUTRATE,MGMUT,MUTVAR,MIGVAR)

REAL B(NDEME),GENO(NDEME,MAXPOP,NLIVE),MUTRATE

REAL MGMUT,MUTVAR,MIGVAR

INTEGER SEED,MCOUNT(NDEME)

INTEGER*4 I,J,K

DO 75 I = 1,NDEME

DO 74 J = 1,MCOUNT(I)

! ****** Mutate the Kth allele of the Jth individual in the Ith deme *****

DO 71 K = 1,NPHALL

IF (RAN(SEED).LT.MUTRATE) THEN

GENO(I,J,K) = GENO(I,J,K) + MUTVAR*ZMUT(SEED)

END IF

71 CONTINUE

IF (MGMUT.EQ.0.0) THEN

GOTO 74

ELSE

DO 77 K = (NPHALL+1),NALL

IF (RAN(SEED).LT.MGMUT) THEN

GENO(I,J,K) = GENO(I,J,K) + MIGVAR*ZMUT(SEED)

IF (GENO(I,J,K).LT.0) THEN

GENO(I,J,K) = 0

END IF

END IF

77 CONTINUE

END IF

74 CONTINUE

75 CONTINUE

RETURN

END

!

!

!

! *********THIS SUBROUTINE DETERMINES THE PHENOTYPE *********

SUBROUTINE DEVELOP(MCOUNT,B,GENO,NDEME,MAXPOP,NALL,NMALL,NPHALL,

C NMEAN,NPLAST,NMIGR,NPHENO,NLIVE)

REAL B(NDEME),GENO(NDEME,MAXPOP,NLIVE),MEAN

INTEGER MCOUNT(NDEME)

INTEGER*4 I,J,K

DO 75 I = 1,NDEME

DO 74 J = 1,MCOUNT(I)

! ******* Compute the individual's phenotype ***********

MEAN = 0

PLAST = 0

PMIG = 0

DO 72 K = 1,NMALL

72 MEAN = MEAN + GENO(I,J,K) !Sum the non-plastic loci

DO 73 K = (NMALL+1),NPHALL

73 PLAST = PLAST + GENO(I,J,K) !Sum the plastic loci

DO 76 K = (NPHALL+1),NALL

76 PMIG = PMIG + GENO(I,J,K) !Sum the migration loci

GENO(I,J,NMEAN) = MEAN

GENO(I,J,NPLAST) = PLAST

GENO(I,J,NMIGR) = PMIG

GENO(I,J,NPHENO) = MEAN + (B(I)*PLAST) !Determine the phenotype

GENO(I,J,NLIVE) = 1.0 !Set to "alive"

74 CONTINUE

75 CONTINUE

RETURN

END

!

!

!

! ****** THIS FUNCTION GENERATES A RANDOM STANDARD NORMAL DEVIATE ******

REAL FUNCTION ZMUT(IDUM)

DATA ISET/0/

IF (ISET.EQ.0) THEN

1 V1 = 2.0*RAN(IDUM) - 1.0

V2 = 2.0*RAN(IDUM) - 1.0

R = V1**2 + V2**2

IF (R.GE.1.0.OR.R.EQ.0.0) GOTO 1

FAC = SQRT(-2.0*LOG(R)/R)

GSET = V1*FAC

ZMUT = V2*FAC

ISET = 1

ELSE

ZMUT = GSET

ISET = 0

ENDIF

RETURN

END

!

!

!

!

! ****** THIS SUBROUTINE MEASURES METAPOPULATION MEAN VALUES *****

SUBROUTINE MEASURE(MCOUNT,GENO,POPMEAN,NDEME,MAXPOP,

C NALL,NMALL,NPALL,NPHALL,NMGALL,NMEAN,

C NPLAST,NMIGR,NPHENO,NLIVE,STS,OPT,NPARAM)

REAL GENO(NDEME,MAXPOP,NLIVE),POPMEAN(NDEME,NPARAM)

REAL STS(NDEME),OPT(NDEME)

DOUBLE PRECISION M1,M2,PL1,PL2,C01,C02,C12,PH1,PH2

DOUBLE PRECISION RSUM,SS,VM,VP,VP2,P21,P22

INTEGER MCOUNT(NDEME)

INTEGER*4 I,J,K

H = -0.5

DO 62 I = 1,NDEME

IF (MCOUNT(I).EQ.0) THEN

DO 67 K = 1,NPARAM

67 POPMEAN(I,K) = 0

GOTO 62

ELSE

XI = MCOUNT(I)

VM1 = 0

M1 = 0

M2 = 0

VPL1 = 0

PL1 = 0

PL2 = 0

VP2 = 0

VMG1 = 0

P21 = 0

P22 = 0

C01 = 0

C02 = 0

C12 = 0

PH1 = 0

PH2 = 0

F1 = 0

! ******* COMPUTE MEANS, SD, AND CORR FOR EACH DEME ********

DO 61 J = 1,MCOUNT(I)

RSUM = 0

SS = 0

! ******* COMPUTE VALUES FOR NON-PLASTIC LOCI ********

DO 64 K = 1,NMALL

RSUM = RSUM + GENO(I,J,K)

SS = SS + GENO(I,J,K)*GENO(I,J,K)

64 CONTINUE

VM = SD(RSUM,SS,NMALL)

RSUM = 0

SS = 0

! ******* COMPUTE VALUES FOR LINEAR PLASTIC LOCI *******

DO 65 K = (NMALL+1),NPHALL

RSUM = RSUM + GENO(I,J,K)

SS = SS + GENO(I,J,K)*GENO(I,J,K)

65 CONTINUE

VPL = SD(RSUM,SS,NPALL)

RSUM = 0

SS = 0

! ******* COMPUTE VALUES FOR MIGRATION LOCI ******

DO 66 K = (NPHALL+1),NALL

RSUM = RSUM + GENO(I,J,K)

SS = SS + GENO(I,J,K)*GENO(I,J,K)

66 CONTINUE

VMG = SD(RSUM,SS,NMGALL)

! ******* COMPUTE DEME SUMS, SSQ, AND CP **********

M1 = M1 + GENO(I,J,NMEAN)

M2 = M2 + GENO(I,J,NMEAN)*GENO(I,J,NMEAN)

VM1 = VM1 + VM

PL1 = PL1 + GENO(I,J,NPLAST)

PL2 = PL2 + GENO(I,J,NPLAST)*GENO(I,J,NPLAST)

VPL1 = VPL1 + VPL

P21 = P21 + GENO(I,J,NMIGR)

P22 = P22 + GENO(I,J,NMIGR)*GENO(I,J,NMIGR)

VMG1 = VMG1 + VMG

C01 = C01 + (GENO(I,J,NMEAN) * GENO(I,J,NPLAST))

C02 = C02 + (GENO(I,J,NMEAN) * GENO(I,J,NMIGR))

C12 = C12 + (GENO(I,J,NPLAST) * GENO(I,J,NMIGR))

PH1 = PH1 + GENO(I,J,NPHENO)

PH2 = PH2 + GENO(I,J,NPHENO)*GENO(I,J,NPHENO)

61 CONTINUE

! ******* COMPUTE MEAN FITNESS *************

DO 63 J = 1,MCOUNT(I)

Z = (GENO(I,J,NPHENO) - OPT(I))/STS(I)

FITNESS = EXP(H * Z * Z)

F1 = F1 + FITNESS

63 CONTINUE

! ******* COMPUTE DEME MEANS, SD AND CORR *********

POPMEAN(I,1) = M1/XI

POPMEAN(I,2) = PL1/XI

POPMEAN(I,3) = P21/XI

POPMEAN(I,4) = CORR(M1,M2,PL1,PL2,C01,MCOUNT(I))

POPMEAN(I,5) = CORR(M1,M2,P21,P22,C02,MCOUNT(I))

POPMEAN(I,6) = CORR(PL1,PL2,P21,P22,C12,MCOUNT(I))

POPMEAN(I,7) = VM1/XI

POPMEAN(I,8) = SD(M1,M2,MCOUNT(I))

POPMEAN(I,9) = VPL1/XI

POPMEAN(I,10) = SD(PL1,PL2,MCOUNT(I))

POPMEAN(I,11) = VMG1/XI

POPMEAN(I,12) = SD(P21,P22,MCOUNT(I))

POPMEAN(I,13) = PH1/XI

POPMEAN(I,14) = SD(PH1,PH2,MCOUNT(I))

POPMEAN(I,15) = F1/XI

END IF

62 CONTINUE

RETURN

END

!

!

!

!

! ****** THIS SUBROUTINE OUTPUTS TRANSIENT VALUES *****

SUBROUTINE TRANSIENT(NSH,MS,GRAD,SLOPE,BIASM,BIAS,MR,

C NGEN,NMLOCI,NPLOCI,NMIGL,LINKAGE,VMLOCI,

C VPLLOCI,MCOUNT,GENO,NDEME,MAXPOP,NMEAN,NPLAST,

C NMIGR,NPHENO,NLIVE,STS,OPT,MEANFIT,ENOISE,COST,CNOISE)

REAL GENO(NDEME,MAXPOP,NLIVE),DMEAN(7),RSUM(7),OUTS(7)

REAL STS(NDEME),OPT(NDEME),MEANFIT,MR,LINKAGE

DOUBLE PRECISION M1,M2,PL1,PL2,C01,C02,C12,PH1

DOUBLE PRECISION P21,P22,ND,ND2,CDM,CDPH,MSUM,PHSUM

INTEGER MCOUNT(NDEME),BIAS,VMLOCI,VPLLOCI

INTEGER*4 I,J,K

H = -0.5

DO 3 K = 1,7

3 RSUM(K) = 0

ND = 0

ND2 = 0

CDM = 0

CDPH = 0

NDEAD = 0

DO 62 I = 1,NDEME

IF (MCOUNT(I).EQ.0) THEN

DO 4 K=1,7

4 DMEAN(K) = 0

NDEAD = NDEAD + 1

GOTO 62

ELSE

XI = MCOUNT(I)

M1 = 0

M2 = 0

PL1 = 0

PL2 = 0

P21 = 0

P22 = 0

C01 = 0

C02 = 0

C12 = 0

PH1 = 0

F1 = 0

! ******* COMPUTE MEANS, SD, AND CORR FOR EACH DEME ********

DO 61 J = 1,MCOUNT(I)

! ******* COMPUTE DEME SUMS, SSQ, AND CP **********

M1 = M1 + GENO(I,J,NMEAN)

M2 = M2 + GENO(I,J,NMEAN)*GENO(I,J,NMEAN)

PL1 = PL1 + GENO(I,J,NPLAST)

PL2 = PL2 + GENO(I,J,NPLAST)*GENO(I,J,NPLAST)

P21 = P21 + GENO(I,J,NMIGR)

P22 = P22 + GENO(I,J,NMIGR)*GENO(I,J,NMIGR)

C01 = C01 + (GENO(I,J,NMEAN) * GENO(I,J,NPLAST))

C02 = C02 + (GENO(I,J,NMEAN) * GENO(I,J,NMIGR))

C12 = C12 + (GENO(I,J,NPLAST) * GENO(I,J,NMIGR))

PH1 = PH1 + GENO(I,J,NPHENO)

61 CONTINUE

! ******* COMPUTE DEME MEANS AND CORR *********

DMEAN(1) = M1/XI

DMEAN(2) = PL1/XI

DMEAN(3) = P21/XI

DMEAN(4) = CORR(M1,M2,PL1,PL2,C01,MCOUNT(I))

DMEAN(5) = CORR(M1,M2,P21,P22,C02,MCOUNT(I))

DMEAN(6) = CORR(PL1,PL2,P21,P22,C12,MCOUNT(I))

DMEAN(7) = PH1/XI

END IF

NDEM = NDEME

DO 8 K = 1,7

8 RSUM(K) = RSUM(K) + DMEAN(K)

ND = ND + I

ND2 = ND2 + (I*I)

CDM = CDM + (I*DMEAN(1))

CDPH = CDPH + (I*DMEAN(7))

62 CONTINUE

! ****** COMPUTE METAPOPULATION MEANS *******

NDEM = NDEME - NDEAD

DO 9 K = 1,7

9 OUTS(K) = RSUM(K)/(NDEM*1.0)

MSUM = RSUM(1)

PHSUM = RSUM(7)

SMEAN = RSLOPE(ND,ND2,MSUM,CDM,NDEM)

SPHENO = RSLOPE(ND,ND2,PHSUM,CDPH,NDEM)

! ****** OUTPUT MEANS FOR Nth GENERATION ******

WRITE (7,92) NSH,MS,NMLOCI,NPLOCI,NMIGL,LINKAGE,VMLOCI,

C VPLLOCI,GRAD,SLOPE,BIASM,BIAS,MR,CNOISE,ENOISE,COST,

C NGEN,MEANFIT,(OUTS(K),K=1,7),SMEAN,SPHENO

92 FORMAT (1X,5I4,1X,F5.2,2I3,1X,F5.1,2X,F5.2,2X,F4.2,

C I3,3(2X,F5.2),1X,F6.4,I7,2X,F7.3,9(2X,F7.3))

RETURN

END

!

!

!

!! ****** THIS FUNCTION COMPUTES A POPULATION STANDARD DEVIATION ****

FUNCTION SD(RSUM,SUMSQ,N)

DOUBLE PRECISION SD,RSUM,SUMSQ,VAR

IF (N.EQ.0) THEN

SD = 0

GOTO 91

END IF

VAR = (SUMSQ - ((RSUM*RSUM)/(N*1.0)))/(N*1.0)

IF (VAR.LE.0) THEN

SD = 0

ELSE

SD = SQRT(VAR)

END IF

91 RETURN

END

!

!

!

! ****** THIS FUNCTION COMPUTES A STANDARD ERROR ******

FUNCTION SE(RSUM,SUMSQ,N)

DOUBLE PRECISION SE,RSUM,SUMSQ,VAR

VAR = (SUMSQ - ((RSUM*RSUM)/(N*1.0)))/(N - 1.0)

IF (VAR.LE.0) THEN

SE = 0

ELSE

SE = SQRT(VAR/(N*1.0))

END IF

RETURN

END

!

!

!

! ******* THIS FUNCTION COMPUTES A CORRELATION *******

FUNCTION CORR(X1,X2,Y1,Y2,CP,N)

DOUBLE PRECISION CORR,X1,X2,Y1,Y2,CP

IF (N.EQ.0) THEN

CORR = 0

GOTO 91

END IF

SSX = X2 - ((X1*X1)/(N*1.0))

IF (SSX.LE.0) THEN

CORR = 0

GOTO 91

END IF

SSY = Y2 - ((Y1*Y1)/(N*1.0))

IF (SSY.LE.0) THEN

CORR = 0

GOTO 91

END IF

CORR = (CP - ((X1 * Y1)/(N*1.0))) / SQRT(SSX * SSY)

91 RETURN

END

!

!

!

! ****** THIS FUNCTION COMPUTES A REGRESSION SLOPE *******

FUNCTION RSLOPE(X1,X2,Y1,CP,N)

DOUBLE PRECISION SLOPE,X1,X2,Y1,CP

IF (N.EQ.0) THEN

CORR = 0

GOTO 92

END IF

SSX = X2 - ((X1*X1)/(N*1.0))

IF (SSX.LE.0) THEN

CORR = 0

GOTO 92

END IF

RSLOPE = (CP - ((X1 * Y1)/(N*1.0))) / SSX

92 RETURN

END

!

!

!

! ****** THIS SUBROUTINE DOES MIGRATION ******

SUBROUTINE MIGRATE(MCOUNT,NCOUNT,GENO,NDEME,MAXPOP,

C NALL,NMIGR,NLIVE)

REAL GENO(NDEME,MAXPOP,NLIVE)

INTEGER MCOUNT(NDEME),NCOUNT(NDEME),SEED

INTEGER*4 I,J,K

! ******* Record current deme sizes ********

DO 81 I = 1,NDEME

81 NCOUNT(I) = MCOUNT(I)

DO 85 I = 1,NDEME

! ******* Determine if Jth individual in Ith deme is to migrate to Kth deme *****

DO 83 J = 1,MCOUNT(I)

IF (GENO(I,J,NLIVE).EQ.0) GOTO 83 !If dead go to next individual

K = I + (INT(GENO(I,J,NMIGR)*ZMUT(SEED))) !Determine where migrating

IF (K.EQ.I) THEN

GOTO 83

ELSE IF (K.LT.1) THEN

K = 1

ELSE IF (K.GT.NDEME) THEN

K = NDEME

ELSE

NCOUNT(K) = NCOUNT(K) + 1 !Increment Kth deme size

DO 82 L = 1,NLIVE

82 GENO(K,NCOUNT(K),L) = GENO(I,J,L) !Migrate individual

GENO(I,J,NLIVE) = 0 !Set individual in Ith deme to "dead"

END IF

83 CONTINUE

85 CONTINUE

DO 86 I = 1,NDEME

86 MCOUNT(I) = NCOUNT(I) !Record new population sizes

RETURN

END

!

!

!

!

! *******THIS SUBROUTINE INPUTS FILE LOCATION INFORMATION***********

SUBROUTINE INOUT

CHARACTER*64 OUTNAME,OV*1

5 WRITE (*,6)

6 FORMAT ('Enter the path and name of the output file: ',$)

READ (*,'(A)') OUTNAME

OPEN (unit=6,file=OUTNAME,status='new',ERR=10)

RETURN

10 WRITE (*,*) 'File already exists.'

11 WRITE (*,*) 'Do you wish to overwrite (Y or N)?'

READ (*,'(A)') OV

IF (OV.EQ.'N'.OR.OV.EQ.'n') THEN

GOTO 5

ELSE IF (OV.EQ.'Y'.OR.OV.EQ.'y') THEN

OPEN (unit=6,file=OUTNAME,status='old')

ELSE

GOTO 11

END IF

RETURN

END

!

!

!

! *******THIS SUBROUTINE INPUTS TRANSIENT FILE LOCATION INFORMATION***********

SUBROUTINE TRANSOUT

CHARACTER*64 OUTNAME,OV*1

5 WRITE (*,6)

6 FORMAT ('Enter the name of the transient output file: ',$)

READ (*,'(A)') OUTNAME

OPEN (unit=7,file=OUTNAME,status='new',ERR=10)

RETURN

10 WRITE (*,*) 'File already exists.'

11 WRITE (*,*) 'Do you wish to overwrite (Y or N)?'

READ (*,'(A)') OV

IF (OV.EQ.'N'.OR.OV.EQ.'n') THEN

GOTO 5

ELSE IF (OV.EQ.'Y'.OR.OV.EQ.'y') THEN

OPEN (unit=7,file=OUTNAME,status='old')

ELSE

GOTO 11

END IF

RETURN

END

!

!

!

! ******* THIS SUBROUTINE WRITES TRANSIENT METADATA ********

SUBROUTINE TRANSMETA(NDEME,GTYPE,PLTYPE,NPOP,MAXPOP,NREP,

C OPTMEAN,OFFSET,WIDTH,MUTRATE,MUTVAR,

C MGMUT,MIGVAR)

CHARACTER GTYPE*4,PLTYPE*4,RUDATE*24

REAL MUTRATE,MUTVAR,MGMUT,MIGVAR

CALL FDATE(RUDATE)

WRITE (7,1)

1 FORMAT ('METADATA')

WRITE (7,2) RUDATE

2 FORMAT ('Date file was generated: ',A24)

WRITE (7,45)

WRITE (7,3)

3 FORMAT ('Simulation parameters:')

WRITE (7,4) NDEME

4 FORMAT ('The number of demes is ',I3)

WRITE (7,7) GTYPE

7 FORMAT ('Type of gradient is ',A4,' (Cont(inuous) or Step)')

WRITE (7,71) PLTYPE

71 FORMAT ('The reaction norm is ',A4,' (Cont(inuous) or Step)')

WRITE (7,8) NPOP

8 FORMAT ('The carry capacity is ',I4)

WRITE (7,9) MAXPOP

9 FORMAT ('The maximum population size after migration is ',I4)

WRITE (7,10) NREP

10 FORMAT ('The number of replicates per parameter set is ',I3)

WRITE (7,11) OPTMEAN

11 FORMAT ('The offset of the gradient along the Y axis is ',F4.1)

WRITE (7,12) OFFSET

12 FORMAT ('The offset of the gradient along the X axis is ',F4.1)

WRITE (7,49) WIDTH

49 FORMAT ('The width of the selection function is ',F5.2)

WRITE (7,17) MUTRATE

17 FORMAT ('The phenotype mutation rate is ',F6.4)

WRITE (7,18) MUTVAR

18 FORMAT ('The standard deviation of phenotype mutation is ',F6.3)

WRITE (7,47) MGMUT

47 FORMAT ('The migration mutation rate is ',F6.4)

WRITE (7,48) MIGVAR

48 FORMAT ('The standard deviation of migration mutation is ',F6.3)

WRITE (7,45)

WRITE (7,19)

19 FORMAT ('The data columns are:')

WRITE (7,72)

72 FORMAT ('1 = Soft/hard selection (1=soft)')

WRITE (7,61)

61 FORMAT ('2 = Move/select order (1=move first)')

WRITE (7,13)

13 FORMAT ('3 = The number of non-plastic loci')

WRITE (7,14)

14 FORMAT ('4 = The number of linear plastic loci')

WRITE (7,16)

16 FORMAT ('5 = The number of migration loci')

WRITE (7,51)

51 FORMAT ('6 = The strength of genetic linkage')

WRITE (7,52)

52 FORMAT ('7 = Initial genetic variation of mean loci')

WRITE (7,53)

53 FORMAT ('8 = Initial genetic variation of plasticity loci')

WRITE (7,20)

20 FORMAT('9 = The slope or step of the optimal phenotype function')

WRITE (7,22)

22 FORMAT ('10 = The slope of the plasticity function')

WRITE (7,50)

50 FORMAT('11 = The bias in the initial non-plastic allelic values')

WRITE (7,23)

23 FORMAT ('12 = The bias in the initial plasticity allelic values')

WRITE (7,24)

24 FORMAT ('13 = The initial migration rate')

WRITE (7,62)

62 FORMAT ('14 = The autocorr of environmental noise at selection')

WRITE (7,63)

63 FORMAT ('15 = The SD of environmental noise at selection')

WRITE (7,73)

73 FORMAT ('16 = The cost of plasticity')

WRITE (7,29)

29 FORMAT ('17-27 = Transient parameters for the first replicate:')

WRITE (7,26)

26 FORMAT ('17 = The generation number')

WRITE (7,27)

27 FORMAT ('18 = Mean fitness across demes')

WRITE (7,30)

30 FORMAT ('19 = Mean of non-plastic loci')

WRITE (7,31)

31 FORMAT ('20 = Mean of plastic loci')

WRITE (7,32)

32 FORMAT ('21 = Mean of migration loci')

WRITE (7,33)

33 FORMAT ('22= Correl of non-plastic & plastic loci')

WRITE (7,34)

34 FORMAT ('23 = Correl of non-plastic & migration loci')

WRITE (7,35)

35 FORMAT ('24 = Correl of plastic & migration loci')

WRITE (7,36)

36 FORMAT ('25 = Mean of net phenotype')

WRITE (7,37)

37 FORMAT ('26 = Slope of non-plastic loci across demes')

WRITE (7,38)

38 FORMAT ('27 = Slope of mean phenotype across demes')

WRITE (7,45)

WRITE (7,44)

44 FORMAT ('THE DATA ARE:')

45 FORMAT (' ')

RETURN

END

!

!

!

! ******* THIS SUBROUTINE WRITES METADATA ********

SUBROUTINE METADATA(NDEME,GTYPE,PLTYPE,NPOP,MAXPOP,NREP,

C TGEN,OPTMEAN,OFFSET,WIDTH,MUTRATE,

C MUTVAR,MGMUT,MIGVAR)

CHARACTER GTYPE*4,PLTYPE*4,RUDATE*24

REAL MUTRATE,MUTVAR,MGMUT,MIGVAR

INTEGER TGEN

CALL FDATE(RUDATE)

WRITE (6,1)

1 FORMAT ('METADATA')

WRITE (6,2) RUDATE

2 FORMAT ('Date file was generated: ',A24)

WRITE (6,45)

WRITE (6,3)

3 FORMAT ('Simulation parameters:')

WRITE (6,4) NDEME

4 FORMAT ('The number of demes is ',I3)

WRITE (6,7) GTYPE

7 FORMAT ('Type of gradient is ',A4,' (Cont(inuous) or Step)')

WRITE (6,71) PLTYPE

71 FORMAT ('The reaction norm is ',A4,' (Cont(inuous) or Step)')

WRITE (6,8) NPOP

8 FORMAT ('The carry capacity is ',I4)

WRITE (6,9) MAXPOP

9 FORMAT ('The maximum population size after migration is ',I4)

WRITE (6,10) NREP

10 FORMAT ('The number of replicates per parameter set is ',I3)

WRITE (6,54) TGEN

54 FORMAT ('The maximum number of generations is ',I7)

WRITE (6,11) OPTMEAN

11 FORMAT ('The offset of the gradient along the Y axis is ',F4.1)

WRITE (6,12) OFFSET

12 FORMAT ('The offset of the gradient along the X axis is ',F4.1)

WRITE (6,49) WIDTH

49 FORMAT ('The width of the selection function is ',F5.2)

WRITE (6,17) MUTRATE

17 FORMAT ('The phenotype mutation rate is ',F6.4)

WRITE (6,18) MUTVAR

18 FORMAT ('The standard deviation of phenotype mutation is ',F6.3)

WRITE (6,47) MGMUT

47 FORMAT ('The migration mutation rate is ',F6.4)

WRITE (6,48) MIGVAR

48 FORMAT ('The standard deviation of migration mutation is ',F6.3)

WRITE (6,45)

WRITE (6,19)

19 FORMAT ('The data columns are:')

WRITE (6,72)

72 FORMAT ('1 = Soft/hard selection (1=soft)')

WRITE (6,61)

61 FORMAT ('2 = Move/select order (1=move first)')

WRITE (6,13)

13 FORMAT ('3 = The number of non-plastic loci')

WRITE (6,14)

14 FORMAT ('4 = The number of linear plastic loci')

WRITE (6,16)

16 FORMAT ('5 = The number of migration loci')

WRITE (6,51)

51 FORMAT ('6 = The strength of genetic linkage')

WRITE (6,52)

52 FORMAT ('7 = Initial genetic variation of mean loci')

WRITE (6,53)

53 FORMAT ('8 = Initial genetic variation of plasticity loci')

WRITE (6,20)

20 FORMAT('9 = The slope or step of the optimal phenotype function')

WRITE (6,22)

22 FORMAT ('10 = The slope of the plasticity function')

WRITE (6,50)

50 FORMAT('11 = The bias in the initial non-plastic allelic values')

WRITE (6,23)

23 FORMAT ('12 = The bias in the initial plasticity allelic values')

WRITE (6,24)

24 FORMAT ('13 = The initial migration rate')

WRITE (6,62)

62 FORMAT ('14 = The autocorr of environmental noise at selection')

WRITE (6,63)

63 FORMAT ('15 = The SD of environmental noise at selection')

WRITE (6,73)

73 FORMAT ('16 = The cost of plasticity')

WRITE (6,74)

74 FORMAT ('17 = Number of population crashes')

WRITE (6,27)

27 FORMAT ('18 = Mean fitness across demes at equilibrium')

WRITE (6,28)

28 FORMAT ('19 = Deme number')

WRITE (6,29)

29 FORMAT ('20-49 = Deme parameters:')

WRITE (6,30)

30 FORMAT ('20 = Mean of non-plastic loci, 21 = SE of those loci')

WRITE (6,31)

31 FORMAT ('22 = Mean of linear plastic loci, 23 = SE')

WRITE (6,32)

32 FORMAT ('24 = Mean of migration loci, 25 = SE')

WRITE (6,33)

33 FORMAT ('26 = Correl of non-plastic & linear plastic, 27 = SE')

WRITE (6,34)

34 FORMAT ('28 = Correl of non-plastic & migration, 29 = SE')

WRITE (6,35)

35 FORMAT ('30 = Correl of linear plastic & migration, 31 = SE')

WRITE (6,36)

36 FORMAT ('32 = SD within-individual non-plastic loci, 33 = SE')

WRITE (6,37)

37 FORMAT ('34 = SD among-individual non-plastic loci, 35 = SE')

WRITE (6,38)

38 FORMAT ('36 = SD within-individual linear plastic loci, 37 = SE')

WRITE (6,39)

39 FORMAT ('38 = SD among-individual linear plastic loci, 39 = SE')

WRITE (6,40)

40 FORMAT ('40 = SD within-individual migration loci, 41 = SE')

WRITE (6,41)

41 FORMAT ('42 = SD among-individual migration loci, 43 = SE')

WRITE (6,42)

42 FORMAT ('44 = Mean of net phenotype, 45 = SE')

WRITE (6,43)

43 FORMAT ('46 = SD of net phenotype, 47 = SE')

WRITE (6,46)

46 FORMAT ('48 = Deme mean fitness, 49 = SE')

WRITE (6,45)

WRITE (6,44)

44 FORMAT ('THE DATA ARE:')

45 FORMAT (' ')

RETURN

END

!

!

!

! ******** THIS SUBROUTINE OUTPUTS MEAN DEME *******

! ******** VALUES FOR EACH PARAMETER SET *******

SUBROUTINE OUTER(NSH,MS,GRAD,SLOPE,MR,OUTS,NDEME,MEANFIT,

C NCRASH,NPARAM,BIASM,BIAS,NMLOCI,NPLOCI,NMIGL,

C LINKAGE,VMLOCI,VPLLOCI,ENOISE,COST,CNOISE)

REAL MR,MEANFIT,OUTS(NDEME,(2*NPARAM)),LINKAGE

INTEGER BIAS,VMLOCI,VPLLOCI

INTEGER*4 I,J

DO 90 I = 1,NDEME

90 WRITE (6,91) NSH,MS,NMLOCI,NPLOCI,NMIGL,LINKAGE,VMLOCI,

C VPLLOCI,GRAD,SLOPE,BIASM,BIAS,MR,CNOISE,

C ENOISE,COST,NCRASH,MEANFIT,I,(OUTS(I,J),J=1,30)

91 FORMAT (1X,5I4,1X,F5.2,2I3,1X,F5.1,2X,F5.2,2X,F4.2,

C I3,3(2X,F5.2),2X,F6.4,1X,I3,2X,F5.3,I4,

C 6(F8.3,F7.3),3(2F9.5,2F7.3),F8.2,5F7.3)

RETURN

END

!

!

!

! ******* THIS SUBROUTINE TELLS YOU WHERE THE PROGRAM IS AT **********

SUBROUTINE WHEREAT(NSH,MS,GRAD,SLOPE,BIASM,BIAS,MR,NGEN,

C MEANFIT,NREP,NMLOCI,NPLOCI,NMIGL,LINKAGE,

C VMLOCI,VPLLOCI,CNOISE,ENOISE,COST)

REAL MR,MEANFIT,LINKAGE

INTEGER BIAS,VMLOCI,VPLLOCI

WRITE (*,92) NSH,MS,NMLOCI,NPLOCI,NMIGL,LINKAGE,VMLOCI,

C VPLLOCI,GRAD,SLOPE,BIASM,BIAS,MR,CNOISE,ENOISE,

C COST,NGEN,MEANFIT,NREP

92 FORMAT (5I2,1X,F4.2,2I3,1X,F4.1,1X,F5.2,1X,F4.2,

C I3,3(1X,F4.2),1X,F6.4,I7,1X,F5.3,I3)

RETURN

END
